# Systems pharmacology using mass spectrometry identifies critical response nodes in prostate cancer

**DOI:** 10.1038/s41540-018-0064-1

**Published:** 2018-07-02

**Authors:** H. Alexander Ebhardt, Alex Root, Yansheng Liu, Nicholas Paul Gauthier, Chris Sander, Ruedi Aebersold

**Affiliations:** 1 Department of Biology, Institute of Molecular Systems Biology, ETH Zürich, Auguste Piccard Hof 1, Zürich, Switzerland; 2 Systems Biology Ireland, University College Dublin, Belfield, Dublin 4, Ireland; 3 Computational Biology Center, Memorial Sloan-Kettering Cancer Center, 1275 York Ave., New York, NY USA; 4 Department of Physiology, Biophysics, and Systems Biology, Weill Cornell Graduate School of Medical Sciences, 1300 York Ave., New York, NY USA; 5 Department of Pharmacology, Yale University School of Medicine, West Haven, CT USA; 6 cBio Center, Dana-Farber Cancer Institute, Boston, MA USA; 7 Department of Cell Biology, Harvard Medical School, Boston, MA USA; 8 Faculty of Science, University of Zürich, Zürich, Switzerland

## Abstract

In the United States alone one in five newly diagnosed cancers in men are prostate carcinomas (PCa). Androgen receptor (AR) status and the PI3K-AKT-mTOR signal transduction pathway are critical in PCa. After initial response to single drugs targeting these pathways resistance often emerges, indicating the need for combination therapy. Here, we address the question of efficacy of drug combinations and development of resistance mechanisms to targeted therapy by a systems pharmacology approach. We combine targeted perturbation with detailed observation of the molecular response by mass spectrometry. We hypothesize that the molecular short-term (24 h) response reveals details of how PCa cells adapt to counter the anti-proliferative drug effect. With focus on six drugs currently used in PCa treatment or targeting the PI3K-AKT-mTOR signal transduction pathway, we perturbed the LNCaP clone FGC cell line by a total of 21 treatment conditions using single and paired drug combinations. The molecular response was analyzed by the mass spectrometric quantification of 52 proteins. Analysis of the data revealed a pattern of strong responders, i.e., proteins that were consistently downregulated or upregulated across many of the perturbation conditions. The downregulated proteins, HN1, PAK1, and SPAG5, are potential early indicators of drug efficacy and point to previously less well-characterized response pathways in PCa cells. Some of the upregulated proteins such as 14-3-3 proteins and KLK2 may be useful early markers of adaptive response and indicate potential resistance pathways targetable as part of combination therapy to overcome drug resistance. The potential of 14-3-3ζ (YWHAZ) as a target is underscored by the independent observation, based on cancer genomics of surgical specimens, that its DNA copy number and transcript levels tend to increase with PCa disease progression. The combination of systematic drug perturbation combined with detailed observation of short-term molecular response using mass spectrometry is a potentially powerful tool to discover response markers and anti-resistance targets.

## Author summary

Metastatic prostate cancer is often treated with pharmacological agents to prevent the tumor from expanding; however, despite advances in drug development patients often die of the disease. An international research team lead by Ruedi Aebersold (ETH Zürich, Switzerland) and Chris Sander (Dana Faber Cancer Institute, Boston, USA) asked how prostate cancer cells adapt to pharmacological treatment on the molecular protein level and find a general response in their prostate cancer model. Next, they asked if similar changes are found in prostate cancer patients. Indeed, the same proteins upregulated in prostate cancer models are also upregulated in prostate cancer patients. Immediately, this has implications for patient treatment stratification and opens new avenues for drug developments in metastatic prostate cancer.

## Introduction

Prostate cancer (PCa) is classified into distinct stages: localized PCa (treated with surgery and/or radiation), metastatic PCa (treated with androgen deprivation therapy (ADT)), castration-resistant PCa (CRPC, treated with second-line therapy), and treatment refractory disease.^1^ The stages largely indicate the course of PCa disease progression and define common clinical interventions during respective stages. In PCa diagnosis and risk assessment pose particular challenges.^2^ Approximately 99% of men who are diagnosed with PCa will not die from PCa but rather die from unrelated medical hazards.^3,4^ However, with 30% of the male population diagnosed with PCa the challenge remains beyond localized PCa and to better understand disease progression. As genomic alterations are common in PCa large cohorts of patients across the entire spectrum of PCa were genotyped and recurrent genomic alterations were identified using the latest DNA sequencing and DNA copy number variation methodologies. These latest efforts largely validated prior studies into genomic instability in PCa,^5–7^ but instead of genomic regions the resolution of the current DNA sequencing methods allow for mutation per gene status reports. Hence, potentially druggable events such as androgen receptor (AR) amplification, ETS fusion, and proteins of the dysregulated PI3K-AKT-mTOR^8^ signal transduction pathway^9^ critical for disease progression were identified. Following these and other leads, drugs targeting products of some of these genes, such as enzalutamide^10^ targeting AR or temsirolimus targeting mTOR, were developed and are in clinical use.

In targeted therapy it is frequently observed that resistance emerges relatively soon after the administration of targeted drugs. For example, it has been observed that tumors frequently express alternative signaling pathways in response to drug treatment, thus making them resistant to the initially applied targeting therapy.^11,12^ Combination therapy inhibiting alternative signaling pathways for additive or even synergistic effect in tumor regression response is a promising strategy to overcome resistance. Carver and co-workers showed that combination therapy targeting both AR and PI3K-AKT-mTOR signaling caused near complete regression in a PTEN-deficient mouse model with PCa.^13^ Against both signaling pathways a number of clinically approved drugs are available.^14^ Schwartz and colleagues took advantage of these pharmacological advances and chose drug combinations against AR and multiple PI3K isoforms and suggested that the largest tumor mass reduction was achieved using a triple drug combination.^15^

Despite these advances, new combination therapies are necessary, possibly targeting molecules beyond AR and PI3K-AKT-mTOR pathways as resistance to AR inhibitors continue to emerge. Developing new combination therapies is challenging, partly due to the vast theoretical search space: screening a natural product library of 20,000 compounds in all triple combinations would result in 1.3 × 10^12^ experiments and is therefore experimentally intractable. Even starting with a chemical compound library comprised of 1000 approved drugs a screen for a triple combination would result in 175 × 10^6^ experiments, which are still too many experiments to carry out for high content screening of phenotypic readouts. In order to reduce the number of initial compounds for screening, Korkut and co-workers used an in silico pathway extraction and reduction algorithm to quantify 143 proteomic/phenotypic entities under 89 perturbation conditions targeting Raf signaling in drug-resistant melanoma cells. The integration of quantitative proteomic/phenotypic data obtained from the pharmaceutical perturbation experiments predicted that inhibition of MYC together with either BRAF or MEK constitute an effective therapeutic approach for RAF-resistant melanoma cells.^16^ This integration of prior knowledge greatly reduced the theoretical search space and resulted in a new combination therapy successfully blocking cell growth.

**Scheme 1:**
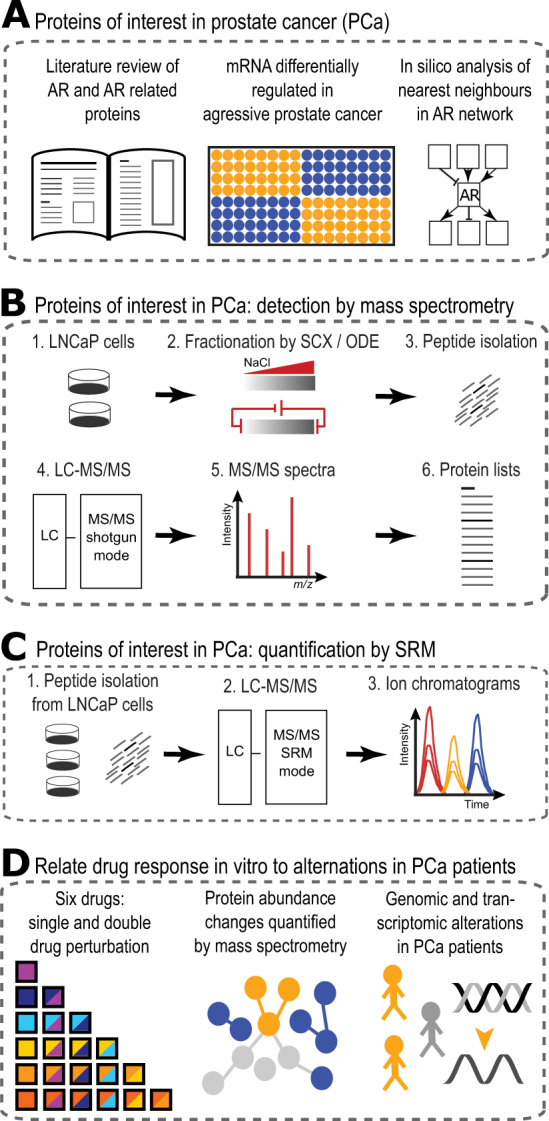
Project overview. **a** A list of proteins of interest in PCa was generated by extensive literature review, comparison of gene expression data, and in silico analysis of nearest neighbor in androgen receptor (AR) pathway. **b** To establish which proteins are identifiable in the LNCaP PCa model, cells were grown in a Petri dish and harvested. From the cell pellets the proteome was extracted and extensively fractionated using strong cation exchange (SCX) chromatography and off-gel electrophoresis (OGE). Fractions were purified and peptides identified using LC-MS/MS in discovery (or shotgun) mode. The MS/MS spectra were annotated to obtain a list of proteins identifiable in LNCaP cells. **c** Quantifying protein abundance following pharmacological treatment was done on undepleted lysate level. Peptides were quantified using targeted proteomics (SRM-MS) and resulting ion chromatograms were analyzed with the software tool Skyline. **d** Six clinically relevant drugs were chosen and all single plus double drug combinations were added to a PCa model. Proteins served as phenotypic readouts of drug response and were quantified using mass spectrometry in selected reaction monitoring (SRM) mode. Immediate protein abundance changes were analyzed across the dataset identifying a group of proteins consistently upregulated. Publicly available DNA alteration and transcriptomics data from PCa patients was analyzed to link immediate response to drug treatment with adaptations found in PCa disease progression

We sought to apply a similar prior knowledge strategy to elucidate immediate protein response to drug treatment of PCa. Instead of quantifying proteins using protein arrays, which require either commercially available high-quality antibodies or the generation of new antibodies, targeted proteomics using LC-MS/MS (liquid chromatography coupled to mass spectrometers) has the potential to quantify proteins across a large sample cohort with a CV of less than 0.1.^17^ Compared to developing new antibodies, development of a targeted proteomics assay is rapid due to available public depositories such as SRMAtlas^18^ and Panorama^19^ containing multiple high-quality targeted assays per protein. Due to the reliability and high sample throughput of targeted proteomics, the technology is mostly applied to clinical research validating protein signatures across large sample cohorts.^20^ However, targeted proteomics has not been applied to systematically quantify a set of proteins in a model system perturbed with small molecule inhibitors. Hence, we sought to apply selected reaction monitoring (SRM), the prototypical targeted proteomics method, to characterize the immediate protein response of an early metastatic PCa model to small molecule inhibitor treatment. We then integrated proteomics with genotypic data from PCa patients (Scheme 1) in order to foster understanding of PCa disease progression. Specifically, we sought to answer several biological questions: (1) Which proteins should be inhibited to achieve a maximal response detected at the level of targeted proteins in response to pharmacological perturbation? (2) Is there a uniform protein response to pharmacological drug perturbation or does each drug and drug combination induce a different protein response in a metastatic PCa model? (3) Can we identify proteins outside the AR and PI3K-AKT-mTOR signal transduction pathways that are important for the cellular response to pharmacological treatment within the AR and PI3K-AKT-mTOR pathways? As PCa model we chose the hormone sensitive LNCaP clone FGC (ATCC® CRL-1740™). The cells were originally isolated from lymph nodes of a PCa patient and represent an early metastatic PCa model prior to true androgen independence.^21^ In respect to the PCa disease spectrum LNCaP cells are past the localized PCa and early metastatic stage but still hormone sensitive which might give clues toward disease progression in PCa. Here we show that there are commonly regulated proteins in LNCaP cells shortly after pharmacological perturbation. Analyzing PCa patient data, some of these commonly regulated proteins show an increase in DNA amplification and RNA expression levels comparing local with metastatic PCa. Taken the short-term response to pharmacological treatment together with the manifestation of increased protein expression as function of PCa disease progression might serve as evidence to develop new treatment targets outside the PI3K-AKT-mTOR/AR pathways in PCa.

## Results

### Proteins of interest in PCa

As the first step of the study we compiled a list of proteins for targeted mass spectrometry in a three-step approach. First, we surveyed the literature and manually curated proteins frequently related to PCa. Second, the literature based set of proteins was supplemented with highly regulated genes (ratio +/−2) in metastatic compared to normal prostate tissue using microarray data.^22^ The literature and experimental data resulted in an initial list of proteins of interest in PCa. Third, we projected the initial list of proteins onto the REACTOME database.^23^ REACTOME contains protein–protein interaction data from a vast number of experiments and predictions across many tissue and disease types. If two proteins on our prostate-specific list were connected by a linker protein in the interaction network (e.g., a protein that both interact with), this linker protein was also added to the list of proteins. This resulted in a curated list of 490 proteins with relevance for PCa that was the starting point for the development of targeted assays (Supplementary Table 1).

### Development of measurement methods for targeted proteomics

**Fig. 1:**
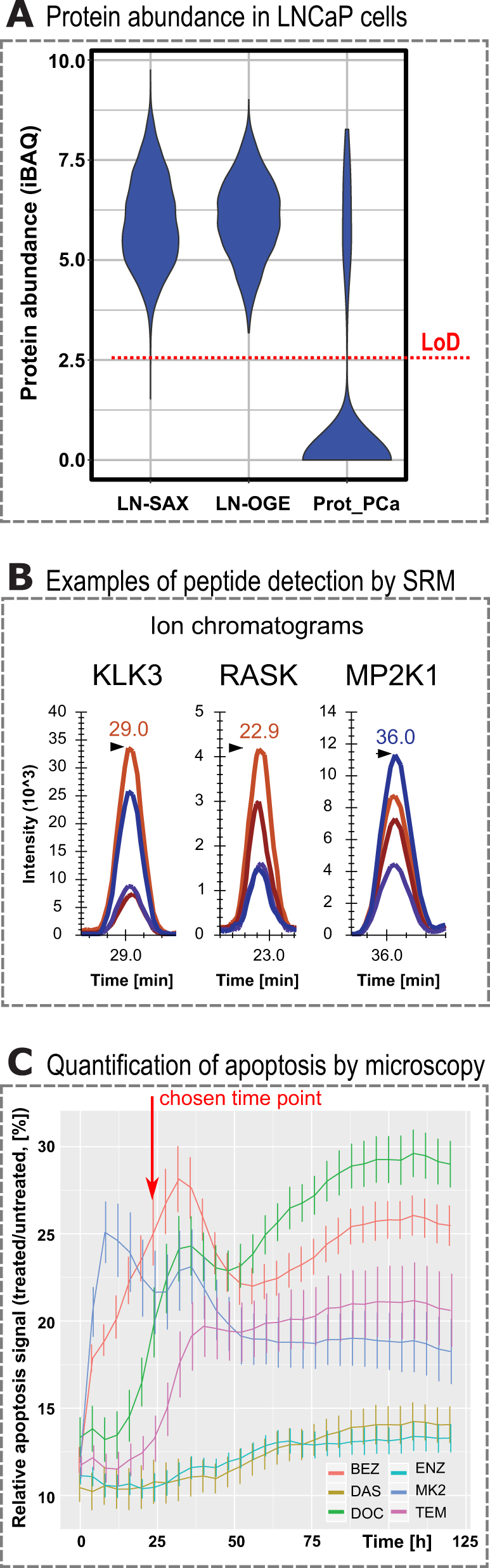
Development of SRM assays and initial drug testing. **a** Total proteome was isolated from LNCaP cells and fractionated using strong anion exchange (LN_SAE^25^). Separately, the LNCaP proteome was fractionated using strong cation exchange and off-gel electrophoresis (LN_OGE). In both cases, all fractions were analyzed using mass spectrometry (LC-MS/MS) and protein abundances calculated (iBAQ units^64^). The limit of detection (LoD) is indicated by a horizontal dashed red line. A quarter of the 490 proteins of interest in prostate cancer (Prot_PCa) are detectable in LNCaP cells using current mass spectrometry equipment. **b** For detectable proteins in LNCaP cell lysate SRM assays were developed. Examples of ion chromatograms show four co-eluting transitions with base line separation for peptides corresponding to KLK3 (prostate-specific antigen), RASK (GTPase KRas), and MP2K1 (MAP kinase kinase 1). **c** LNCaP cells were incubated with a caspase 3/7 fluorescent probe and six clinically relevant drugs (BEZ: NVP-Bez235, DAS: dasatinib, DOC: docetaxel, ENZ: enzalutamide, MK2: MK2206, TEM: temsirolimus). As a function of time the fluorescent signal corresponding to the degree of apoptosis was quantified by an automated fluorescent microscope. The ratio of treated to untreated apoptosis signal shows a large dynamic range at 24 h, a time point we chose for subsequent perturbation experiments

As the second step of the study we generated definitive mass spectrometric targeted assays for the quantification of the 490 proteins or a subset thereof. To achieve this goal we (a) determined which of the 490 proteins were detectable in LNCaP cells and (b) developed targeted proteomics assays for the detectable proteins using standard procedures.^24^ To determine detectability, we used bottom-up shotgun proteomics to analyze an extensively fractionated LNCaP lysate. The total tryptic peptide sample generated from the cell lysate was fractionated by off-gel electrophoresis (isoelectric focusing) or strong cation exchange (SCX) chromatography. From these fractions we cumulatively identified 110 of the 490 proteins which cover the whole dynamic range in LNCaP cells as determined by Geiger and co-workers^25^ (Fig. 1a). Additionally, targeted assays representing proteins on the 490 target protein set were identified from exploratory perturbation experiments in LNCaP cells and collected from a publicly available database.^26^ Having accumulated targeted assays from various sources, we set out to determine which of these assays led to the unambiguous detection of the respective target protein in an unfractionated cell lysate prepared from LNCaP clone FGC cells, using a triple quadruple mass spectrometer (AB Sciex Triple Quad 5500 LC-MS/MS) in targeted proteomics mode (Multiple or SRM). Per peptide, at least four transitions were measured and in certain cases stable isotope-labeled peptides were spiked into the peptide mix to confirm peptide retention times (Fig. 1b). For SRM assay development ranging from SRM spectral library over setting up SRM methods to analyzing primary SRM mass spectrometry data Skyline was used, which is a widely used open source document editor for creating and analyzing targeted proteomics experiments.^27^ These measurements established that peptides representing 52 proteins were consistently quantifiable in whole cell lysate of LNCaP cells by SRM (Supplementary Fig. 1). These 52 proteins cover mainly small GTPase-mediated signal transduction and transmembrane receptor protein tyrosine kinase signal transduction pathways, including the PI3K-ATK-mTOR pathway (see Supplementary Fig. 2, Supplementary Table 2).

### Initial phenotypic screening

Having established proteomic parameters to quantify a set of target proteins, we next selected a PCa model for pharmacological perturbations. As PCa model we chose LNCaP cells, specifically LNCaP clone FGC. The cells are responsive to male hormone 5α-dihydrotestosterone, express endogenously both NKX3.1 and AR, and harbors a truncated PTEN at position 340 (403 full length).^28,29^ As LNCaP clone FGC is both androgen sensitive and progresses toward AR-independent growth, this cell line model is ideally positioned to test a wide range of small molecule inhibitors used in PCa.

As pharmacological agents for our perturbation experiments we chose four small molecule inhibitors targeting the PI3K-AKT-mTOR pathway^30^: dasatinib (DAS, *c*_final_ = 100 nM) targeting Proto-oncogene tyrosine-protein kinase Src,^31^ NVP-Bez235 (BEZ, *c*_final_ = 500 nM), which acts dually on mTOR and phosphatidylinositol 4,5-bisphosphate 3-kinases,^32^ MK-2206 (MK2, *c*_final_ = 1 µM) targeting RAC-alpha serine/threonine-protein kinase Akt1/2/3,^33^ and temsirolimus (TEM, *c*_final_ = 100 nM) targeting Serine/threonine-protein kinase mTOR.^34^ Further, we chose docetaxel (DOC, *c*_final_ = 10 nM),^35^ a chemotherapy drug inhibiting microtubule depolymerization, and the antiandrogen enzalutamide (ENZ, *c*_final_ = 10 µM) inhibiting AR activity.^10,36^ All six drug concentrations were chosen based on community consensus concentrations reported in the literature.

Having selected both biological system and the pharmacological perturbants, we set out to determine a reasonable time point for quantifying the perturbed proteome. To this end we quantified apoptosis via a fluorescence-based assay in a time-dependent manner using an automated microscope. Perturbing LNCaP clone FGC cells with the six drugs individually resulted in distinct apoptosis signals as function of time (Fig. 1c). The strongest induction of an apoptotic response was detectable at about 37 h post treatment with MK2 and BEZ. In contrast DAS and DOC showed a low level of apoptotic signal induction over the whole timeline tested. The largest difference in apoptosis signal between treatment conditions was measured around 24 h. Besides apoptosis signals measured by fluorescent microscopy, we quantified 19 proteins (surrogates of the 490 protein list, spanning the entire dynamic range of protein abundance in LNCaP clone FGC) using targeted proteomics as function of time post treatment with ENZ. We found that at 6 h of treatment there was an initial small response to the pharmacological perturbation while at 24 h protein abundance changes were much greater compared to the earlier time point (Skyline file: 1st_time_course_MDV.sky). From our initial experiments quantifying phenotypic parameters of apoptosis and proteomic response we rationalized that 24 h would be a suitable time point to quantify the target protein set by SRM across a systematic, large-scale perturbation matrix in LNCaP cells.

### Drug perturbation matrix

**Fig. 2:**
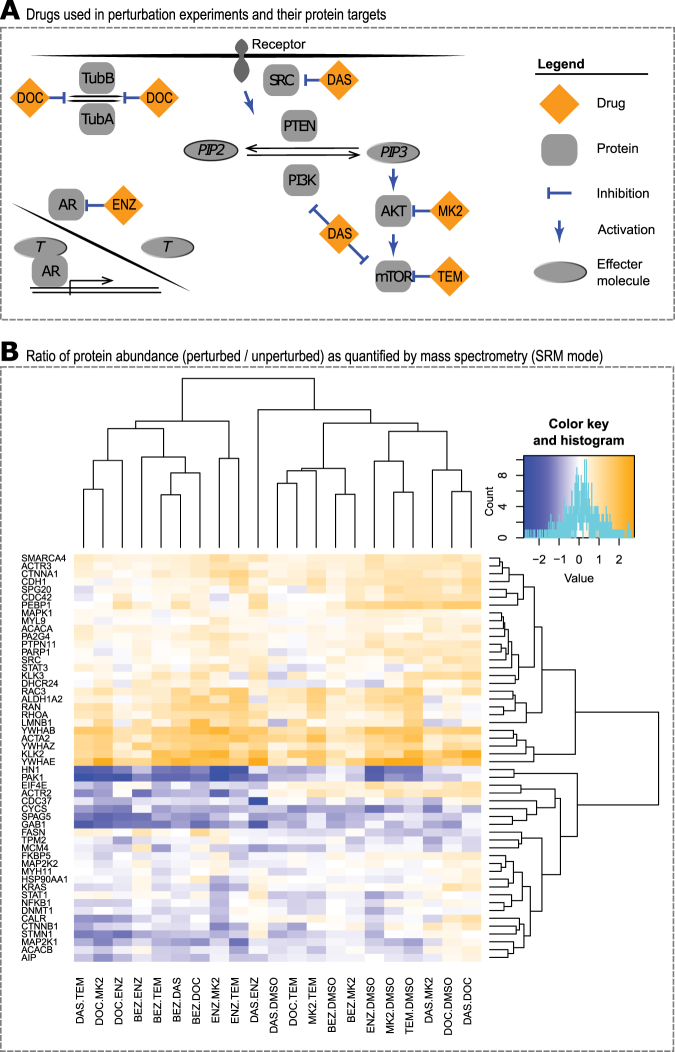
In vitro perturbation experiments: results. **a** Clinically relevant drugs (abbreviations see caption of Fig. 1) focus on interfering with AR and PI3K-AKT-mTOR pathways. Docetaxel was chosen due to its widespread use in treating prostate cancer. **b** Protein abundances were quantified by mass spectrometry in SRM mode, log2 transformed and relative protein ratios (treated/untreated) calculated. Unsupervised clustering of these protein values per condition across the entire perturbation matrix resulted in clusters of proteins largely upregulated and proteins largely downregulated as short-term response to drug treatment. Protein names are standard gene names

Following the selection of a PCa system, six clinically relevant drugs (Fig. 2a), and an initial screen of these drugs to establish a reasonable time point we carried out our systems pharmacology study to characterize the proteome under perturbation. For these experiments, we seeded two million cells in a 10 cm Petri dish and carried out perturbations in single and all pairwise drug combinations, resulting in 21 treatment conditions plus vehicle control. Following drug perturbation for 24 h, cells were harvested and the proteome extracted. Following trypsinization of the proteome and desalting, peptides were subjected to LC-MS/MS in targeted proteomics mode (SRM) using the 77 established targeted assays. The resulting ion chromatograms were analyzed by Skyline to obtain the area under the curve per analyte and condition. At this stage, proteins with a CV > 0.4 across biological replica of the same condition were omitted from further analysis. Next, data was further analyzed by MSStats for statistical significance analysis. To visualize the results of 52 protein abundance patterns across the entire perturbation matrix, we plotted the ratio of treatment condition to untreated (vehicle control). Figure 2b shows the resulting heatmap in which proteins with a higher abundance compared to vehicle control are depicted in yellow, while proteins with a lower abundance compared to vehicle control are depicted in blue. The heatmap shows two general trends: some drug perturbation are less effective than others in changing protein abundance. Second, there are sets of proteins that were consistently downregulated or upregulated under most perturbations. These two observations are discussed in more detail below.

### Single vs. double drug combinations

**Fig. 3:**
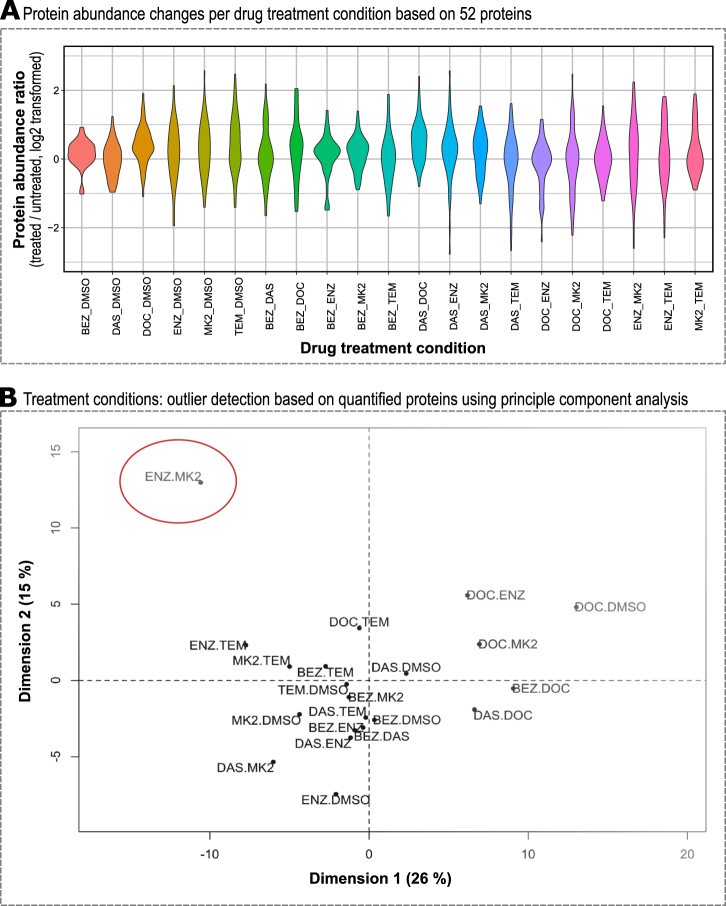
Global protein profile analysis. **a** Protein abundances were log2 transformed and ratios between drug treated and untreated calculated. There are treatment conditions with little change in protein abundance as a result of treatment, e.g., BEZ or BEZ + MK2, while other treatment conditions have a profound effect on short-term protein abundance changes following treatment, e.g., DAS + ENZ or ENZ + MK2 (for drug abbreviations, see caption of Fig. 1). **b** Principle component analysis (PCA) of protein abundance changes groups DOC treatment conditions and identifies ENZ + MK2 as distinct double treatment condition (red circle)

To systematically compare which drug combinations altered protein abundance more strongly than others we visualized protein abundance changes using violin plots which show both the amplitude of change and distribution across the response range (see Fig. 3). Some single drug treatment conditions showed very minute changes in abundance of proteins quantified, such as BEZ or DAS, whereas ENZ, MK2, and TEM showed larger changes in the single treatment condition. The violin plots for double drug treatment condition showed non-uniform behavior: some double drug combinations such as BEZ and MK2 showed small effects on the quantified proteome, while other double drug combination such as ENZ and MK2 changed abundance of nearly all quantified proteins. There was a trend toward small changes in proteome when the applied drugs targeted components of the same signal transduction pathway (e.g., BEZ + MK2 or DAS + MK2). In contrast, we observed larger protein abundance changes of nearly all proteins quantified when the applied drugs targeted components of different signal transduction pathways (e.g., DAS + ENZ or ENZ + MK2).

To better identify commonalities and differences in proteome response we carried out a principle component analysis (PCA) across all perturbation conditions (see Fig. 3b). In this cluster analysis, DOC containing perturbations clustered together which might indicate a consistent and dominant effect of DOC on the proteome under the conditions tested. The great majority of conditions clustered around the center of the PCA plot with the exception of ENZ + MK2 which caused the quantified proteome to alter distinctively from all other treatment conditions tested with 49 of 52 proteins showing statistically significant changes of *p* < 0.05 (Supplementary Fig. 3). Based on our proteomics data, ENZ + MK2 double treatment had the largest effect on the quantified proteome in LNCaP cells.

### Strongest protein responders

**Fig. 4:**
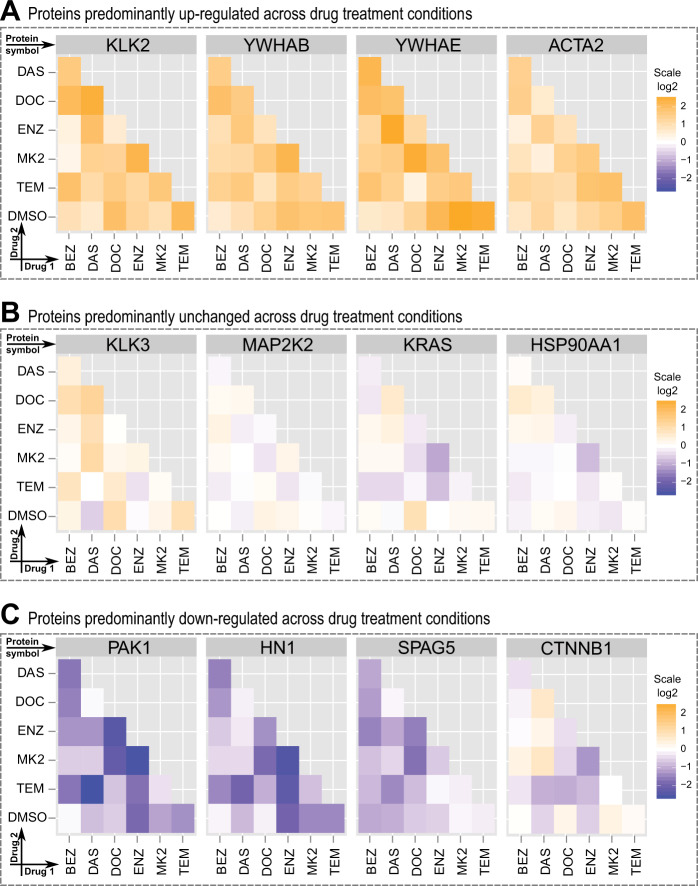
Protein centric view of abundance ratio across conditions. **a** Predominantly upregulated proteins such as enzymes (e.g., KLK2) and chaperons (e.g., 14-3-3 proteins) as short-term response to pharmacological conditions tested in LNCaP cells. **b** Frequently investigated proteins in PCa (e.g., KLK3 [PSA] or HSP90) show little change in protein abundance across the tested pharmacological perturbations in LNCaP cells. **c** Predominantly down-regulated proteins as short-term response to pharmacological conditions tested. Proteins such as PAK1 and HN1 are poorly characterized in PCa

Besides the broad analysis of conditions using violin plots and PCA analysis, we carried out a protein centric analysis across all treatment conditions, both in terms of protein abundance changes (Supplementary Fig. 4) and statistical significance (Supplementary Fig. 5). This protein centric analysis revealed strongly responding proteins with significant upregulation or downregulation in response to most drug treatments. Proteins presenting with a higher abundance across many treatment conditions were KLK2 and all quantified 14-3-3 isoforms (Fig. 4a). We hypothesize that these upregulated proteins are a common short-term response to pharmacological treatment in PCa cells. Our results warrant further mechanistic studies into these proteins in PCa as we rationalize that a downregulation or inhibition of these upregulated proteins (Fig. 4a) might increase the effectiveness of a given pharmacological intervention, e.g., ENZ + MK2.

Surprisingly, many well-known cancer proteins such as KRAS, KLK3 (prostate-specific antigen, PSA), and HSP90 did not exhibit significant changes in the immediate response after 24 h post pharmacological treatment (Fig. 4b). There were, however, proteins less commonly studied in PCa such as HN1 and PAK1 which significantly decreased in protein abundance in most of the treatment conditions (Fig. 4c). Downregulation of PAK1 was shown to inhibit PCa cell migration and microinvasion via modulation of matrix metalloproteinase 9 and various growth factors.^37^ Downregulation of HN1 was reported using an AKT inhibitor^38^ and an increase in HN1 levels increases migration in a PCa cell line model.^39^ Taken these facts together, decreases in HN1 and PAK1 protein abundance levels offer an early indication of drug effectiveness and inhibits PCa migration. We reason that these proteins may serve as early markers of efficacy in treated patients, as these proteins are far more responsive after 24 h compared to KLK3 (PSA). Our protein centric analysis of the quantitative proteomics data obtained following short-term (24 h) drug perturbation in the PCa model LNCaP clone FGC revealed several highly regulated proteins. How do these in vitro results correlate with PCa patient data? To answer this question for upregulated proteins we shifted our attention to genomic datasets containing PCa patient data.

### Patient data integration

**Fig. 5:**
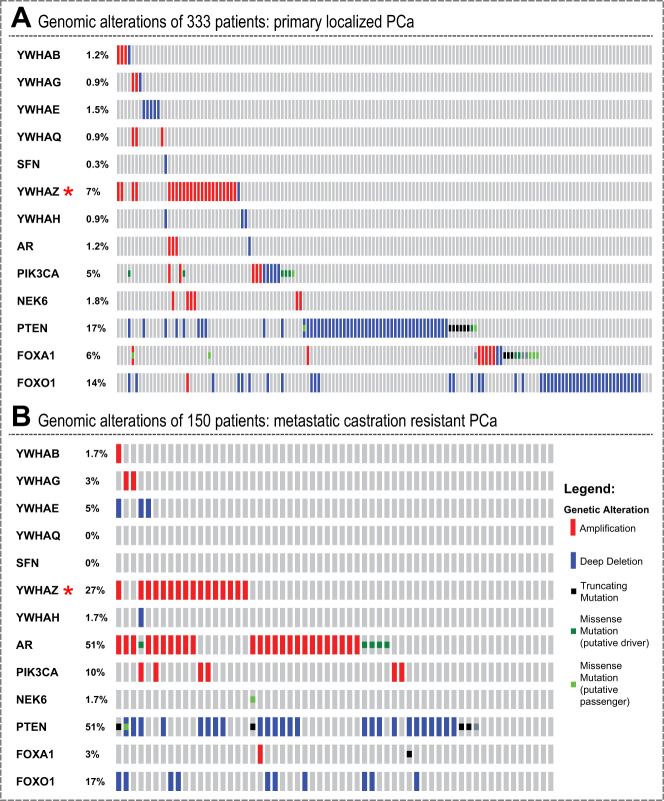
Genomic alterations in PCa patients. **a** Genomic alterations of 333 primary PCa specimens. Each bar represents a patient. In blue are deep gene deletions, while in red are gene amplifications. Clearly visible are three sub-groups: FOXO1 deletions (14%), PTEN deletions (17%), and YWHAZ amplifications (7%, indicated by red asterisks). **b** Genomic alterations of an independent 150 metastatic CRPC querying the same genes as in **a**. Consistent with prior knowledge is the AR gene amplification in 51% of patient samples and PTEN deletion in 51% of cases. YWHAZ is amplified in 27% of metastatic castration-resistant PCa, a four-fold increase over primary localized PCa patients

**Fig. 6:**
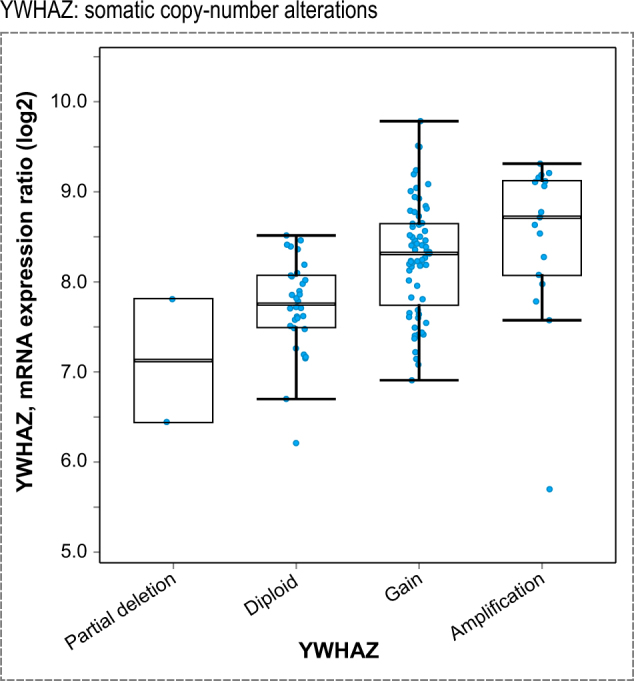
Expression profiles of PCa patients. In patients with partial deletion of the YWHAZ loci, mRNA levels are lower compared to regular diploid mRNA expression levels. Upon gain or amplification of the YWHAZ loci more mRNA is transcribed. Together with the correlation of increased YWHAZ amplification as function of disease progression (see Fig. 5) data suggests YWHAZ as driver of PCa disease progression in many patients

Amplification, deletion or mutation events of genes per patient are available. If upregulation of proteins is important for cancer cell survival, we hypothesize that certain clonal events might benefit from encoding this upregulation genetically. Gene amplification, together with increased mRNA expression, is one way to increase protein abundance in a cell. Hence, we analyzed nucleic acid molecular abnormalities in primary and metastatic PCa patient datasets. We observed that in a cohort of 333 primary PCa patients 14-3-3 proteins are amplified or deleted in 12.7% of patient tumors.^40^ The frequency of this nucleic acid abnormality is similar to the common PTEN (17%) and FOXO1 (14%) deletions in the same dataset (Fig. 5a). Focusing on 14-3-3, PTEN, and FOXO1, we performed detailed genetic analysis of 150 advanced PCa samples (metastatic CRPC).^41^ In this second dataset, 14-3-3 proteins were amplified or deleted in 38.4% of patient samples, while PTEN and FOXO1 were deleted or mutated in 51 and 17% of cases, respectively (Fig. 5b). Of the 14-3-3 proteins, 14-3-3ϛ (YWHAZ) alone accounted for 7% of all 14-3-3 amplifications in the primary samples and for 27% in the metastatic PCa dataset. This four-fold increase in frequency of YWHAZ amplification between primary and metastatic PCa is higher than the three-fold increase of PTEN deletion. As YWHAZ is located on chromosome 8q a recurrent hot spot for amplification in PCa the argument of a passenger mutation arises. To distinguish amplifications that are drivers from passengers, calculating correlation between copy number and mRNA expression can be useful.^42^ Driver alterations tend to show positive correlation between degree of copy number amplification and mRNA expression; passenger alterations show no correlation. Comparison of median copy number with median mRNA expression shows a positive correlation (Fig. 6), suggesting that YWHAZ is a driver alteration. Therefore, we conclude that our in vitro proteomics data of a PCa model correlates with observations made in genomic data of PCa patients in the case of YWHAZ. We assume that patients in the cohort were treated with the standard of care, i.e., ADT. Therefore, the upregulation of YWHAZ may be associated with resistance to ADT. It may also be associated with disease progression. Established cell line models have similar patterns: the LNCaP cell line does not have an amplification of 8q (Supplementary Table 4); however, the commonly used metastatic PCa cell line PC3 has amplifications in NCOA2, MYC, and YWHAZ, which is suggestive of an 8q amplification. As abundance of YWHAZ rises both as a result of pharmacological treatment in vitro and as function of disease progression from local to metastatic CRPC we postulate that the increase in YWHAZ with drug treatment is indicative of adaptive response, i.e., potential resistance mechanism, with potential implications for the design of anti-resistance combination therapy.

## Discussion

### Protein list detectable by targeted proteomics in LNCaP

PCa is frequently described as being driven by AR and PI3K-AKT-mTOR signal transduction pathways. To better understand this network of proteins in PCa during pharmacological treatment, we reviewed the literature, analyzed differentially expressed micro-array data of PCa patient samples, and computationally scrutinized the AR and PI3K-AKT-mTOR protein neighborhoods resulting in a list of 490 proteins. Recently, 10,000 proteins were identified using current high mass accuracy mass spectrometry technology across many proteomics samples originating from various human tissues and in vitro perturbation experiments.^43^ Cross-referencing these two datasets resulted in 388 proteins detectable in at least one human tissue type. Of the non-detected proteins are transient proteins such as G1/S-specific cyclin-E1 or membrane bound Receptor tyrosine-protein kinase erbB-4. A further reduction in detectable proteins was due to choosing a single cell line. Of the 388 proteins only approximately 110 are detectable in LNCaP cells by our own extensive proteome fractionation. For our targeted proteomics quantification across 22 treatment conditions, we chose 52 proteins which showed a CV of less than 0.4 between biological replica of the same condition. Although the number of proteins is roughly 1/10 of the original 490 list, both protein lists show similar coverage of biological processes when projected onto the REACTOME pathway database.^44^ Hence, these 52 proteins represent sentinels that indicate the state of a wide range of biological processes robustly detectable in LNCaP cells. Nevertheless, interpreting whether changes in protein abundance represent desirable drug effects or resistance mechanisms is challenging.

### 14-3-3 and protein network resistance

Network robustness is one of the five types of resistance mechanisms described by Glickman and Sawyers, and it typically operates on short-term timescales through feedback loops and other mechanisms that maintain oncogenic driver signaling.^45^ Using a systems pharmacology approach we perturbed a metastatic PCa model using small molecule inhibitors against AR and PI3K-AKT-mTOR signal transduction pathways. Our data suggests a key role of 14-3-3 proteins in balancing the AR and PI3K-AKT-mTOR protein network under perturbation. 14-3-3 protein family members interact with a large spectrum of proteins modulating signal transduction.^46^ An increase in 14-3-3 protein abundance signals increased need for modulating additional signal transduction pathways. This increased requirement for stabilizing additional signal transduction pathways presumably results from an increase in transcription/translation of signal transduction proteins requiring 14-3-3 scaffold proteins following pharmacological intervention. Hence, in our model system 14-3-3 protein abundance changes might be used as readout of “drug shock” response, analogous to “heat shock” proteins.

The interplay between AR and PI3K-AKT-mTOR pathway resulting in network robustness was demonstrated by several research groups. For example, Carver and co-workers describe a reciprocal feedback inhibition model whereby short-term inhibition AR or PI3K signaling causes rapid activation of the other pathway through upregulation of transcriptional targets FKBP5 or kinase feedback through mTOR.^13^ Besides elucidating short-term response and communication between two signal transduction pathways, network robustness can also be achieved by redundancy of key players, e.g., PI3K and its isoforms. Schwartz and co-workers showed that inhibiting multiple PI3K isoforms leads to greater tumor mass reduction compared to dual drug treatment targeting AR and predominantly on PI3K isoform.^15^ In this context, our data suggests that 14-3-3 protein isoforms are plausible new target for combinational therapy in PCa. As 14-3-3 proteins are consistently upregulated across many pharmacological perturbations (drug shock proteins (DSP)), decreasing the amount of functional 14-3-3 proteins might increase effectiveness of the treatment.

### Integration with genomic PCa patient data

Besides short-term response on the molecular phenotype level, drug resistance in PCa manifests itself in the long term on the genomic level. Visakorpi and co-workers were first to show an increase in AR amplification as function of disease progression in PCa.^47^ This is not the first example where upregulation of protein expression following drug perturbation over short timescales is associated with DNA amplification in patients that occurs during ADT over long timescales. According to the reciprocal feedback inhibition model, AR expression increases following short-term drug perturbation of PI3K signaling and AR amplification is observed in patients who receive ADT. Hence, if 14-3-3 amplification was not only a short-term adaptation but also an advantage for long-term survival of cancer cells, 14-3-3 amplification will manifest itself on the genomic level. Indeed, we find that there is a four-fold increase in occurrence of 14-3-3ϛ (YWHAZ) amplification between primary and metastatic CRPCa (Fig. 5). To place the four-fold increase into perspective in PCa disease progression, Barbieri and co-workers identified a plausible PCa subtype consisting of mutant SPOP in combination with a lack of ETS family gene rearrangements.^48^ In the Barbieri datasets, the increase of mutant SPOP from early to metastatic CRPCa is two-fold (6–15%). Besides, the genomic amplification transcriptional levels of YWHAZ are also increased (Fig. 6). These observations raise the possibility that YWHAZ amplifications is a driver amplification contributing to disease progression. Further support comes from a study of 213 men who underwent radical prostatectomy. In this patient cohort high levels of YWHAZ strongly associated with high Gleason score and higher risk of fast disease progression to CRPC.^49^ Hence, we hypothesize that the increase in YWHAZ with drug treatment is indicative of adaptive response, i.e., potential resistance mechanism, with potential implications for the design of anti-resistance combination therapy.

Our data show that it was not only YWHAZ that increased in abundance in response in nearly all drug treatments tested, but all other 14-3-3 proteins quantified increased in abundance too. Of all 14-3-3 isoforms, why does YWHAZ manifest itself as being amplified at the genomic level? It is plausible that YWHAZ’s genomic location on chromosome 8q22 contributes to its amplification during disease progression. The entire chromosome 8q region was identified as a genetic hot spot for gene amplification in PCa.^5,9^ The oncogene MYC resides on 8q24,^50^ but it is not known why 8q amplification spans beyond the MYC loci. Hence, researchers explored other genes residing on 8q and established functional links in PCa. For example, Menon et al. knocked down YWHAZ and PTK2 and found that both proliferation and invasion decreased.^51^ Silva et al. found that another gene on 8q, an AR co-activator NCOA2, is associated with aggressive PCa and has elevated mRNA expression.^52^ Therefore, it is likely that several genes on 8q are important in PCa progression and may even have cooperative effects.

Further mechanistic studies are required to elucidate the mechanism by which 14-3-3 proteins act as DSP and the transition from molecular phenotype change upon perturbation to genetic phenotype alteration. Intriguingly, 14-3-3 proteins were consistently found in extracellular vehicles of PCa patients with a Gleason grade 8, which lead Panagopoulos and co-workers to postulate that 14-3-3 proteins are associated with disease progression.^53^ This observation and data presented here support our hypothesis that 14-3-3 proteins are involved in PCa disease progression, possibly via promoting an escape from pharmacological treatment mechanism. Future mechanistic studies might help categorize 14-3-3 protein aided resistance mechanism into restored AR signaling, AR bypass signaling, and/or complete AR independence.^12^

### Markers for early response to treatment

Besides proteins such as 14-3-3 that were consistently upregulated, we detected several proteins that were consistently downregulated across the treatments (Fig. 4). These include PAK1 and HN1. Both proteins show similar behaviors across our pharmacological perturbations. HN1 is part of the AR-AKT signal transduction pathway maintaining the balance between AR localization and PI3K-AKT signaling.^54^ Consistent with previous research, HN1 abundance is largely unaffected by docetaxel (a microtubule depolymerization inhibitor) and most decreased by dual inhibition using MK-2206 (AKT inhibitor) + enzalutamide (antiandrogen). Further research is needed to establish HN1 and/or PAK1 as plausible diagnostic markers for early drug response in patients treated with targeted therapy.

## Conclusion

Our systems pharmacology perturbation of a PCa model coupled with targeted proteomics quantification of proteins as molecular phenotypes offer new data on common short-term response at the cellular level. We hypothesize that the short-term responses reveal adaptive mechanisms that influence the long-term, clinically relevant response and this may point to new therapeutic targets. We also point to a connection between molecular changes seen in the adaptive response to treatment and those observed in progression to more aggressive disease. Thus, proteins consistently upregulated or downregulated after perturbation offer new mechanistic insights into PCa biology and disease progression. We anticipate that larger mass-spectrometry profiling of drug response, which is becoming more accessible and affordable, coupled with computational pathway analysis methods, will be a very useful tool in future applications of systems pharmacology to cancer.

## Methods

### Small molecule inhibitors

We chose four small molecule inhibitors targeting the PI3K-AKT-mTOR pathway, which play a critical role in PCa^30^: dasatinib (*c*_final_ = 100 nM) targeting Proto-oncogene tyrosine-protein kinase Src,^31^ NVP-Bez235 (*c*_final_ = 500 nM), which acts dually on mTOR and phosphatidylinositol 4,5-bisphosphate 3-kinases,^32^ MK-2206 (*c*_final_ = 1 µM) targeting RAC-alpha serine/threonine-protein kinase Akt1/2/3,^33^ and temsirolimus (*c*_final_ = 100 nM) targeting serine/threonine-protein kinase mTOR.^34^ Further, we chose docetaxel (*c*_final_ = 10 nM),^35^ a chemotherapy drug inhibiting microtubule formation, and antiandrogen enzalutamide (*c*_final_ = 10 µM) inhibiting AR activity.^10,36^ Drug concentrations were chosen based on concentrations reported in the literature. For single and double drug combinations experimental set up was as follows in Supplementary MM1.

### Cell culture conditions

LNCaP cells were originally derived from metastatic site (supraclavicular lymph node) nearly four decades ago.^55^ LNCaP clone FGC (ATCC® CRL-1740™) is an androgen and estrogen receptor expressing cell line which does not express *PTEN* due to a deleted allele and a mutated allele. The defined clone available from American Type Culture Collection (Manassas, VA, USA) kept below 20 passages and grown in 10 cm petri dishes in a 37 °C incubator with 5% CO_2_ using RPMI-1640 medium supplemented with 10% BSA, 2 mM L-glutamine, 100 units/mL Penicillin, and 100 µg/mL Streptomycin. For SILAC, LNCaP cells were grown to near confluence in dialyzed medium with either unlabeled or stable isotope-labeled amino acids (Arg ^13^C_6_^15^N_4_, Lys ^13^C_6_^15^N_2_). For the pilot experiment, an enzalutamide concentration of 20 μM was chosen. For the perturbation matrix of six drugs carried out in biological triplicate, cells were treated with 0.5 µM NVP-Bez235, 0.1 µM dasatinib, 0.01 µM docetaxel, 10 µM enzalutamide, 1 µM MK-2206, 0.1 µM temsirolimus, and DMSO as vehicle control. Drug concentrations were kept constant for the dual drug treatment. For sample preparation for proteomic profiling two million cells were seeded in a 10 cm dish and left to settle overnight. Drugs were pipetted directly into the media without a media change and prepared in biological triplicate. After 24 h of drug treatment, dishes were placed on ice, media was aspirated, and cells were washed with 1× PBS (phosphate buffered saline) three times, scraped and pipetted into 1.5 mL tubes. Cells pellets were stored at −80 °C until further use.

### Phenotype quantitation by time-course microscopy: image acquisition

LNCaP cells were plated in a plastic bottom 96-well plate at 5000 cells per well in 100 µL of media and allowed to settle overnight. Drug solutions were prepared in 100 µL of media and added directly to each well without a media change. An IncuCyte ZOOM automated multi-well plate microscope, situated in a 37 °C incubator with 5% CO_2_ with 2015A software was used to acquire live images in two channels–phase contrast and green fluorescent images every 4 h post drug treatment. The IncuCyte ZOOM hardware and software acquired images with a 10× Nikon objective of 1392 × 1040 pixels @ 1.22 µm/pixel in both channels, with four images acquired per well from the well center. Throughout the time-course images are acquired from approximately the same location in the well. For detection of Caspase 3 or 7 activity, a fluorogenic substrate probe was used with a substrate concentration of 5 µM according to manufacturer’s protocol (Biotium NucView(™) 488 Caspase-3 from Biotium, Hayward, CA, USA) with excitation wavelength: 460 nM; passband: [440, 480] nM; and emission wavelength: 524 nM; passband: [504, 544] nM. The green fluorescent channel used an acquisition time of 400 ms. A Dual Color Module 4459 filter module was used.

### Phenotype quantitation by time-course microscopy: image analysis

IncuCyte ZOOM 2015A software was used for auto scaling, background subtraction, thresh holding, segmentation, and area quantification. The software requires the user to set values for these parameters by creating a Processing Definition from several training images. Six training images were chosen by examining many images by eye to represent the spectrum of responses. Parameters were tweaked interactively in order to get reasonably looking background subtraction, thresh holding, and segmentation in each channel. This processing definition is then run automatically over all the images. Data consisting of an average and standard error of Percent Cell Area from the phase channel and Percent Apoptotic Area from the green channel were exported for further normalization, summarization, and visualization in R. The trapezoidal rule was used to summarize the time-course data as area under curve up to 24 h. These AUC values were normalized by computing the ratio of each treatment to vehicle treatment. Plots were made using the ggplot2 package in R.

### OGE and SCX proteome fractionation

Pelleted non-perturbed LNCaP cells were lysed using LysisBuffer (8 M urea, 0.1 M ammonium bicarbonate, 0.1% RapiGest) and nucleic acids sheared using sonication. Non-soluble fraction was precipitated by centrifugation of the sample at 16,100 rcf for 10 min in an Eppendorf centrifuge 5415. Supernatant was analyzed for protein content using BCA assay (Pierce, Fisher Scientific, Perbio Science Switzerland SA, Lausanne, Switzerland). 2 mg of protein were reduced using TCEP (Tris-2-carboxyethyl-phophine, 5 mM final concentration) at 37 °C for 30 min and Cys alkylated using iodoacetamide (10 mM final concentration) at 25 °C for 30 min. Proteins were digested in 2 M urea, 0.1 M ammonium bicarbonate, 0.1% RapiGest buffer using sequence grade trypsin (V511C, Promega, Dübendorf, Switzerland) in a ratio of 1:100 (enzyme:substrate) at 37 °C overnight. Peptide mixture was purified using standard C18 clean up procedure and ½ the material separated on a SCX column. SCX chromatography was carried out on an Agilent 1100 mL flow HPLC using a PolySULFOETHYL ATM column (dimensions: 150 × 1.0 mm, column material bead size: 5 μm with 200 Å pore size). Base liquid phase was comprised of 10 mM KH_2_PO_4_ and 25% acetonitrile. The linear gradient buffer B further contained 0.7 M KCl with a flow rate was at 1 mL/min. Elution profile was monitored with absorbance spectrum at 214 nM and peptides collected in 1 mL fractions. According to the elution profile, peptides were pooled into eight fractions and C18 purified to remove salts. The second half of the peptide mixture generated was fractionated using off-gel fractionation (OGE). OGE was carried out using an Agilent 3100 Off-Gel Fractionator and IPG strips (pH 3–10, 24 cm, GE Healthcare). The strips were prepared according to manufacturer’s manual. The peptide mixture was separated for 50,000 Vhrs (8000 V, 50 µA, 200 mW). Eight fractions were pooled from the OGE separation and C18 purified.

### LC-MS/MS discovery mode

LC-MS/MS was performed using a one-dimensional (1D) chromatographic separations by a nanoLC ultra 2Dplus system (Eksigent, AB SCIEX Germany GmbH, Darmstadt, Germany) coupled to a hybrid ion trap-Orbitrap mass spectrometer with dual-pressure linear ion trap technology (LTQ Orbitrap Velos, Thermo Fisher Scientific Inc., Waltham, MA, USA) as specified in ref. ^56^ Effectively, the LC-MS/MS analysis of all fractions required about 2 days of machine time. Acquired data was converted into mgf format using msconvert version 3.0.3703 and peptide-spectrum matches were annotated by mascot version 2.4 (Matrix Science Ltd.) with the following settings: canonical reviewed Homo sapiens protein database (UniProt, 2015), static modification: carbamindomethyl (Cys), variable modification: oxidation (Met), MS1 mass error: 20 ppm, MS2 mass error: 0.6 Da.

### SRM assay development

First, we fractionated peptides using SCX and OGE obtaining 2873 protein IDs (at 1% FDR), of which 92 overlap with the 490 list. Second, to obtain protein IDs only detected upon AR inhibition, LNCaP cells were pharmacologically perturbed using antiandrogen enzalutamide and the resulting unfractionated proteome analyzed by shotgun LC-MS/MS resulting in 1450 protein IDs (at 1% FDR), of which 45 proteins overlap with the 490 list. In all of the 490 proteins in the initial list, there were 97 proteins identified in our shotgun LC-MS/MS experiments. The information of ionizable peptides, their respective MS/MS spectra, and retention time were used to develop SRM assays. The SRM assay list was extended by eight proteins detected by Kim and coworkers.^57^ Next, the established SRM assays from extensively fractionated lysate were tested for detectability in whole cell lysate. This SRM assay development step took multiple rounds of writing SRM methods, measuring analytes using AB Sciex QTRAP® 5500 (see details below), evaluating peak quality using software package Skyline, selecting best performing peptides, distributing peptides evenly over the LC profile and testing the new SRM method. To validate peptides, stable isotope labeling by amino acids in cell culture (SILAC) was applied to LNCaP cells. Of the 110 proteins from the SRM assay library, 69 proteins were confirmed in a digest of unfractionated lysate with the criteria of precise co-eluting unlabeled (endogenous) and stable isotope-labeled (reference) peptides (Supporting Data SRM initial perturbation). Figure 1b shows the sum of transitions per endogenous or reference peptide as ion chromatograms of KLK3 (iBAQ = 7.5), RASK (iBAQ = N/A), and MP2K1 (iBAQ = 7.1), all with peaks base line separated and precisely co-eluting peak groups with more examples shown in Supplementary Fig. 1a. For maximal quantification accuracy, SRM assays should be free of interfering signals.^58^ A good proxy for estimating interferences is to compare the relative intensities of transitions between endogenous and reference peptides which is implemented as ratio-dot-product in Skyline.^59,60^ Only peptide pairs with a ratio-dot-product of greater than 0.96 were considered further (Supplementary Fig. 1b). Based on positive shotgun identification of fractionated peptides using in silico search engines, co-elution of endogenous and reference peptides in SRM-MS mode, above 0.96 ratio-dot-products, base line separation of individual SRM transitions and very good linear correlation between measured and theoretical retention time (Supplementary Fig. 1c), we conclude that these SRM assays are valid. Further, SRM-MS also enabled quantification of proteins in whole cell lysate not detected in extensively fractionated proteome samples of the same cell line using a shotgun LC-MS/MS approach, which might be due to analytical challenges of extensive fractionation and associated loss of peptides, different LC-MS/MS instrumentation ionizing different subsets of peptides, and perturbed vs. unperturbed cell lines, or a combination of all three factors mentioned.

### Whole cell lysate protocol

The protein amount isolated was normalized by BCA assay prior to trypsin. Peptide amounts were adjusted based on absorbance at 220 nM measurements and 1 μg of endogenous peptides mixed with 1 μg of stable isotope-labeled peptides (SILAC). For the pharmacological perturbation matrix we adjusted the purification method to include a HILIC (hydrophilic interaction chromatography) purification step in order to remove neutral lipids.^61^ The step-by-step protocol is given in the Supplementary MM2.

### LC-MS/MS details targeted proteomics (SRM mode)

A reference spectral library was built from all resulting mascot search results using Skyline^59,60^ with a library build cut-off score of 0.9. Targeted SRM measurements were carried out using a hybrid triple quadrupole/ion trap mass spectrometer (AB Sciex QTRAP® 5500, AB Sciex Switzerland, Brugg, Switzerland) equipped with a nanoelectrospray ion source. 1D chromatographic separations of peptides were performed by a nanoLC ultra 2Dplus system (Eksigent, AB Sciex Switzerland) as detailed in previous work.^58^ The linear LC gradient was 45 min; together with injection and washing of column time, the entire LC method took 1 h 15 min per sample. SRM ion chromatograms were visualized using Skyline^27^ with peak areas per transition for three biological replica (Supplementary Table 3), and were statistically analyzed using MSStats.^62^

MSStats: The R package MSstats v2.3.5 was used to explore the data, summarize multiple biological and technical replicates at the peptide-level data to protein-level, and normalize drug treatment to vehicle treatment.^63^ MSstats fits a mixed-effects linear model that partitions the variance in protein intensity into technical, biological, and experimental components.^62^ To perform summarization the dataProcess function was used with these arguments: logTrans = 2, normalization = “constant”, betweenRunInterferenceScore = FALSE. To perform normalization and statistical testing in comparison with DMSO vehicle treatment using the groupComparision function with arguments set as: labeled = FALSE, scopeOfBioReplication = “restricted”, scopeOfTechReplication = “expanded”, interference = FALSE, featureVar = TRUE, missing.action = “nointeraction”.

### Exploratory data visualizations

To obtain network enrichment analysis REACTOME analysis tool was used. For protein–protein interaction STRING v.10 was queried for the SRM list and filtered to high confidence, experimentally verified interactions. All figures were assembled using INKSCAPE.

### Data availability

Proteomic datasets are available in their respective depositories: MassIVE for discovery data and Panorama for targeted proteomics data.

Discovery data: MassIVE ID: MSV000081956 (total size: 16.59 GB).

Primary SRM data: initial perturbation (https://panoramaweb.org/targetedms/Systems%20Biology%20Ireland%20-%20Ebhardt%20lab/LNperturb/LNperturb_init/showPrecursorList.view?id=27052).

Primary SRM data: full perturbation matrix (https://panoramaweb.org/targetedms/Systems%20Biology%20Ireland%20-%20Ebhardt%20lab/LNperturb/LNperturb_matrix/showPrecursorList.view?id=27196).

## Electronic supplementary material

Skyline output MSStats input file

Copy number alterations for cell lines

Supplementary Tables, Figures and Materials

## Electronic supplementary material

**Supplementary information** accompanies the paper on the *npj Systems Biology and Applications* website (10.1038/s41540-018-0064-1).
