# Supplementary material for: Systems pharmacology using mass spectrometry identifies critical response nodes in prostate cancer
Source: NPJ Syst Biol Appl. 2018 Jul 2;4:26. doi: 10.1038/s41540-018-0064-1 (PMC6026592; doi:10.1038/s41540-018-0064-1)
Supplement: Supplementary file 3 — Supplementary Tables, Figures and Materials [file 41540_2018_64_MOESM3_ESM.pdf]

Supplementary Table 1: List of 490 proteins. A list of proteins of interest in PCa with UniProtID, Symbol, EntrezId and Description.

Uniprot ID	Entry name	Protein names	Official gene name
O00170	AIP_HUMAN	AH receptor-interacting protein	AIP
O00763	ACACB_HUMAN	Acetyl-CoA carboxylase 2	ACACB
O94788	AL1A2_HUMAN	Retinal dehydrogenase 2	ALDH1A2
P01116	RASK_HUMAN	GTPase KRas	KRAS
P06730	IF4E_HUMAN	Eukaryotic translation initiation factor 4E	EIF4E
P07288	KLK3_HUMAN	Prostate-specific antigen	KLK3
P07900	HS90A_HUMAN	Heat shock protein HSP 90-alpha	HSP90AA1
P07951	TPM2_HUMAN	Tropomyosin beta chain	TPM2
P09874	PARP1_HUMAN	Poly [ADP-ribose] polymerase 1	PARP1
P12830	CADH1_HUMAN	Cadherin-1	CDH1
P12931	SRC_HUMAN	Proto-oncogene tyrosine-protein kinase Src	SRC
P16949	STMN1_HUMAN	Stathmin	STMN1
P19838	NFKB1_HUMAN	Nuclear factor NF-kappa-B p105 subunit	NFKB1
P20151	KLK2_HUMAN	Kallikrein-2	KLK2
P20700	LMNB1_HUMAN	Lamin-B1	LMNB1
P24844	MYL9_HUMAN	Myosin regulatory light polypeptide 9	MYL9
P26358	DNMT1_HUMAN	DNA (cytosine-5)-methyltransferase 1	DNMT1
P27797	CALR_HUMAN	Calreticulin	CALR
P28482	MK01_HUMAN	Mitogen-activated protein kinase 1	MAPK1
P30086	PEBP1_HUMAN	Phosphatidylethanolamine-binding protein 1	PEBP1
P31946	1433B_HUMAN	14-3-3 protein beta/alpha	YWHAB
P33991	MCM4_HUMAN	DNA replication licensing factor MCM4	MCM4
P35221	CTNA1_HUMAN	Catenin alpha-1	CTNNA1
P35222	CTNB1_HUMAN	Catenin beta-1	CTNNB1
P35749	MYH11_HUMAN	Myosin-11	MYH11
P36507	MP2K2_HUMAN	Dual specificity mitogen-activated protein kinase kinase 2	MAP2K2
P40763	STAT3_HUMAN	Signal transducer and activator of transcription 3	STAT3
P42224	STAT1_HUMAN	Signal transducer and activator of transcription 1-alpha/beta	STAT1
P49327	FAS_HUMAN	Fatty acid synthase	FASN
P51532	SMCA4_HUMAN	Transcription activator BRG1	SMARCA4
P60763	RAC3_HUMAN	Ras-related C3 botulinum toxin substrate 3	RAC3
P60953	CDC42_HUMAN	Cell division control protein 42 homolog	CDC42
P61158	ARP3_HUMAN	Actin-related protein 3	ACTR3
P61160	ARP2_HUMAN	Actin-related protein 2	ACTR2
P61586	RHOA_HUMAN	Transforming protein RhoA	RHOA
P62258	1433E_HUMAN	14-3-3 protein epsilon	YWHAE
P62736	ACTA_HUMAN	Actin, aortic smooth muscle	ACTA2
P62826	RAN_HUMAN	GTP-binding nuclear protein Ran	RAN
P63104	1433Z_HUMAN	14-3-3 protein zeta/delta	YWHAZ
P99999	CYC_HUMAN	Cytochrome c	CYCS
Q02750	MP2K1_HUMAN	Dual specificity mitogen-activated protein kinase kinase 1	MAP2K1

Q06124	PTN11_HUMAN	Tyrosine-protein phosphatase non-receptor type 11	PTPN11
Q13085	ACACA_HUMAN	Acetyl-CoA carboxylase 1	ACACA
Q13153	PAK1_HUMAN	Serine/threonine-protein kinase PAK 1	PAK1
Q13451	FKBP5_HUMAN	Peptidyl-prolyl cis-trans isomerase FKBP5	FKBP5
Q13480	GAB1_HUMAN	GRB2-associated-binding protein 1	GAB1
Q15392	DHC24_HUMAN	Delta(24)-sterol reductase	DHCR24
Q16543	CDC37_HUMAN	Hsp90 co-chaperone Cdc37	CDC37
Q8N0X7	SPART_HUMAN	Spartin	SPART
Q96R06	SPAG5_HUMAN	Sperm-associated antigen 5	SPAG5
Q9UK76	JUPI1_HUMAN	Jupiter microtubule associated homolog 1	JPT1
Q9UQ80	PA2G4_HUMAN	Proliferation-associated protein 2G4	PA2G4

Supplementary Table 2: Pathway enrichment analysis. All quantified 52 proteins were analysed using REACTOME pathway enrichment analysis tool REACTOME. The enriched pathways are listed together with their respective statistical significance values.

Pathway identifier	Pathway name	Entities pValue	Entities FDR
R-HSA-195258	RHO GTPase Effectors	5.27E-14	3.70E-11
R-HSA-194315	Signaling by Rho GTPases	1.78E-12	6.24E-10
R-HSA-9006934	Signaling by Receptor Tyrosine Kinases	1.13E-11	2.64E-09
R-HSA-5625740	RHO GTPases activate PKNs	4.53E-11	7.92E-09
R-HSA-162582	Signal Transduction	5.33E-10	7.46E-08
R-HSA-6802948	Signaling by high-kinase activity BRAF mutants	9.01E-10	9.67E-08
R-HSA-5663202	Diseases of signal transduction	9.67E-10	9.67E-08
R-HSA-5674135	MAP2K and MAPK activation	1.86E-09	1.30E-07
R-HSA-6802946	Signaling by moderate kinase activity BRAF mutants	1.86E-09	1.30E-07
R-HSA-6802955	Paradoxical activation of RAF signaling by kinase inactive BRAF	1.86E-09	1.30E-07
R-HSA-6802949	Signaling by RAS mutants	1.26E-08	7.96E-07
R-HSA-422475	Axon guidance	1.41E-08	8.17E-07
R-HSA-1643685	Disease	2.12E-08	1.13E-06
R-HSA-2682334	EPH-Ephrin signaling	2.68E-08	1.34E-06
R-HSA-6802952	Signaling by BRAF and RAF fusions	2.93E-08	1.35E-06
R-HSA-4420097	VEGFA-VEGFR2 Pathway	4.01E-08	1.73E-06
R-HSA-5675221	Negative regulation of MAPK pathway	7.03E-08	2.88E-06
R-HSA-194138	Signaling by VEGF	7.88E-08	2.99E-06
R-HSA-3928662	EPHB-mediated forward signaling	9.34E-08	3.36E-06
R-HSA-1433557	Signaling by SCF-KIT	1.23E-07	4.27E-06
R-HSA-1266738	Developmental Biology	1.29E-07	4.27E-06
R-HSA-6802957	Oncogenic MAPK signaling	1.44E-07	4.46E-06
R-HSA-5673000	RAF activation	2.27E-07	6.80E-06
R-HSA-1227986	Signaling by ERBB2	4.47E-07	1.30E-05
R-HSA-449147	Signaling by Interleukins	4.75E-07	1.33E-05
R-HSA-186763	Downstream signal transduction	5.53E-07	1.44E-05
R-HSA-445355	Smooth Muscle Contraction	1.17E-06	2.93E-05
R-HSA-5637810	Constitutive Signaling by EGFRvIII	1.27E-06	2.93E-05
R-HSA-5637812	Signaling by EGFRvIII in Cancer	1.27E-06	2.93E-05
R-HSA-1059683	Interleukin-6 signaling	1.27E-06	2.93E-05
R-HSA-5654743	Signaling by FGFR4	2.84E-06	5.81E-05
R-HSA-5654741	Signaling by FGFR3	2.84E-06	5.81E-05
R-HSA-5683057	MAPK family signaling cascades	2.87E-06	5.81E-05
R-HSA-177929	Signaling by EGFR	3.18E-06	5.81E-05
R-HSA-5637815	Signaling by Ligand-Responsive EGFR Variants in Cancer	3.23E-06	5.81E-05
R-HSA-1236382	Constitutive Signaling by Ligand-Responsive EGFR Cancer Variants	3.23E-06	5.81E-05

R-HSA-1643713	Signaling by EGFR in Cancer	3.23E-06	5.81E-05
R-HSA-5627117	RHO GTPases Activate ROCKs	3.23E-06	5.81E-05
R-HSA-187037	NGF signalling via TRKA from the plasma membrane	3.57E-06	6.07E-05
R-HSA-166520	Signalling by NGF	3.74E-06	6.37E-05
R-HSA-5674499	Negative feedback regulation of MAPK pathway	4.60E-06	7.65E-05
R-HSA-5627123	RHO GTPases activate PAKs	4.78E-06	7.65E-05
R-HSA-445144	Signal transduction by L1	4.78E-06	7.65E-05
R-HSA-1280215	Cytokine Signaling in Immune system	6.12E-06	9.18E-05
R-HSA-112409	RAF-independent MAPK1/3 activation	8.06E-06	1.21E-04
R-HSA-5654736	Signaling by FGFR1	8.69E-06	1.30E-04
R-HSA-163765	ChREBP activates metabolic gene expression	1.08E-05	1.52E-04
R-HSA-447115	Interleukin-12 family signaling	1.23E-05	1.72E-04
R-HSA-5218920	VEGFR2 mediated vascular permeability	1.28E-05	1.79E-04
R-HSA-168256	Immune System	1.36E-05	1.89E-04
R-HSA-186797	Signaling by PDGF	1.45E-05	1.89E-04
R-HSA-6783589	Interleukin-6 family signaling	1.47E-05	1.91E-04
R-HSA-3928663	EPHA-mediated growth cone collapse	1.69E-05	2.19E-04
R-HSA-8876493	InlA-mediated entry of Listeria monocytogenes into host cells	2.10E-05	2.52E-04
R-HSA-112411	MAPK1 (ERK2) activation	2.10E-05	2.52E-04
R-HSA-373755	Semaphorin interactions	2.14E-05	2.57E-04
R-HSA-5626467	RHO GTPases activate IQGAPs	2.18E-05	2.62E-04
R-HSA-109581	Apoptosis	2.77E-05	3.06E-04
R-HSA-180292	GAB1 signalosome	2.78E-05	3.06E-04
R-HSA-2029480	Fcgamma receptor (FCGR) dependent phagocytosis	3.33E-05	3.66E-04
R-HSA-5357801	Programmed Cell Death	3.33E-05	3.66E-04
R-HSA-5663213	RHO GTPases Activate WASPs and WAVEs	3.90E-05	4.29E-04
R-HSA-75035	Chk1/Chk2(Cds1) mediated inactivation of Cyclin B:Cdk1 complex	4.56E-05	5.02E-04
R-HSA-5654738	Signaling by FGFR2	5.12E-05	5.12E-04
R-HSA-5684996	MAPK1/MAPK3 signaling	5.60E-05	5.60E-04
R-HSA-6806834	Signaling by MET	5.78E-05	5.78E-04
R-HSA-5655302	Signaling by FGFR1 in disease	5.85E-05	5.85E-04
R-HSA-111447	Activation of BAD and translocation to mitochondria	6.96E-05	6.96E-04
R-HSA-109606	Intrinsic Pathway for Apoptosis	7.72E-05	7.57E-04
R-HSA-1295596	Spry regulation of FGF signaling	8.41E-05	7.57E-04

Supplementary Table 3: Skyline output of perturbation matrix. All transitions quantified as function of treatment condition. This table served as input for MSStats.

Supplementary Table 3 = separate csv file

Supplementary Table 4: Copy number alterations for cell lines. The cBioPortal was queried for DNA copy number alterations of YWHAZ, MYC and NCOA2 of all available cell line models. Prostate cancer models are highlighted with yellow background.

Supplementary Table 4 = separate csv file

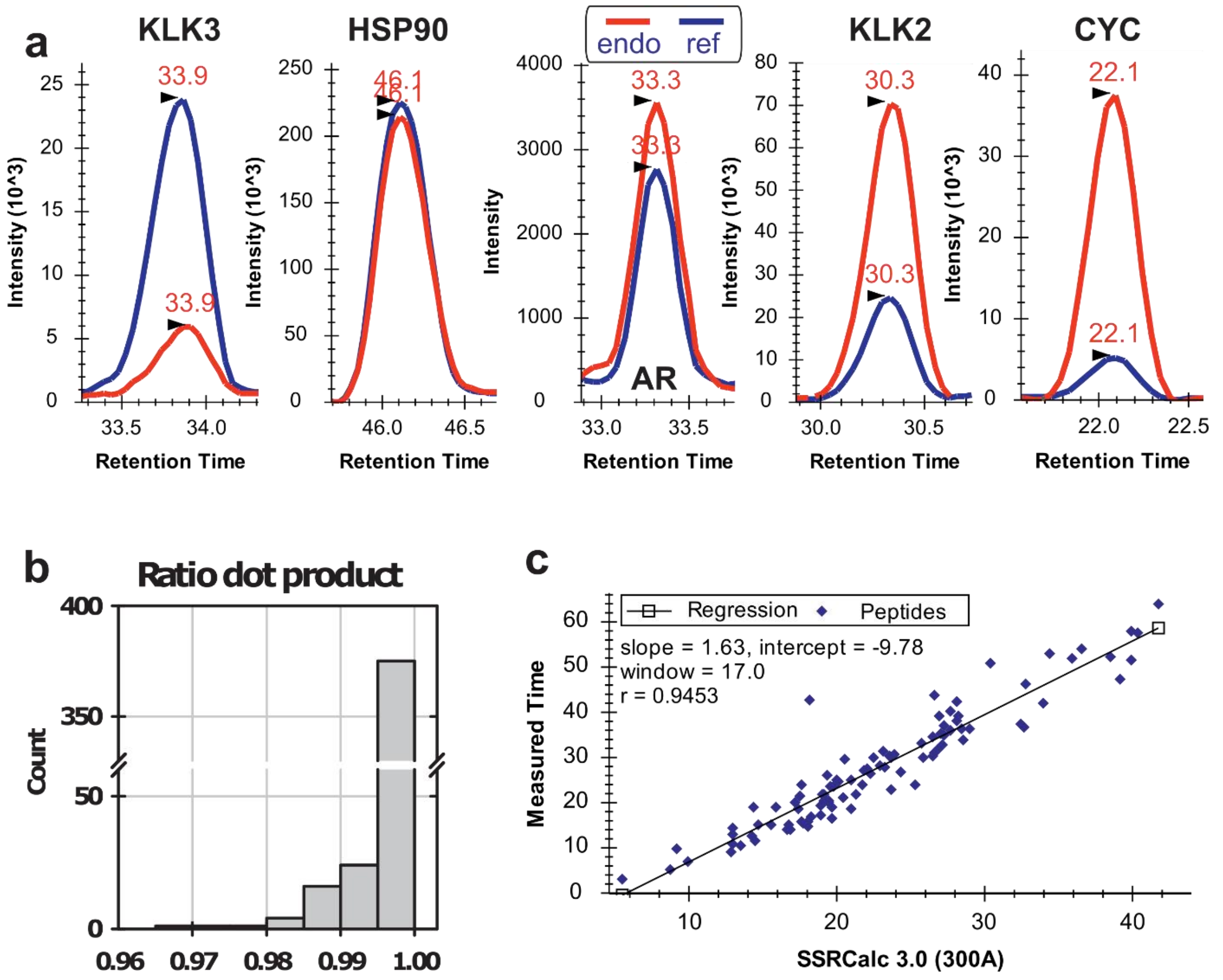

Supplementary Figure 1: SRM assay development. Panel a: Ion chromatograms of the sum of all transitions per peptide which are base line separated from other signals measured. In blue lines the sum of all transition of SILAC labelled peptides while in red are ion chromatograms of endogenous unlabelled peptides isolated from LNCaP cells. Both blue and red lines co-elute which adds further evidence for high confidence SRM assays. Protein names given are official gene names. **Panel b**: The ratio-dot-product is a mathematical description of how similar both SILAC labelled and non-labelled peptide fragments elute, e.g. relative ratio of transitions measured. A score of 1 means identical pattern in both SILAC and non-labelled transitions. The great majority of peptides have a ratio-dot-products of greater than 0.99 indicative of high quality SRM assays. **Panel c**: Measured retention time is plotted as a function of theoretically calculated retention time SSRCalc 3.0 (as function of primary amino acid sequence of the peptide). A linear regression line has a very good r value (r = 0.95) further supporting the notion of high quality SRM assays.

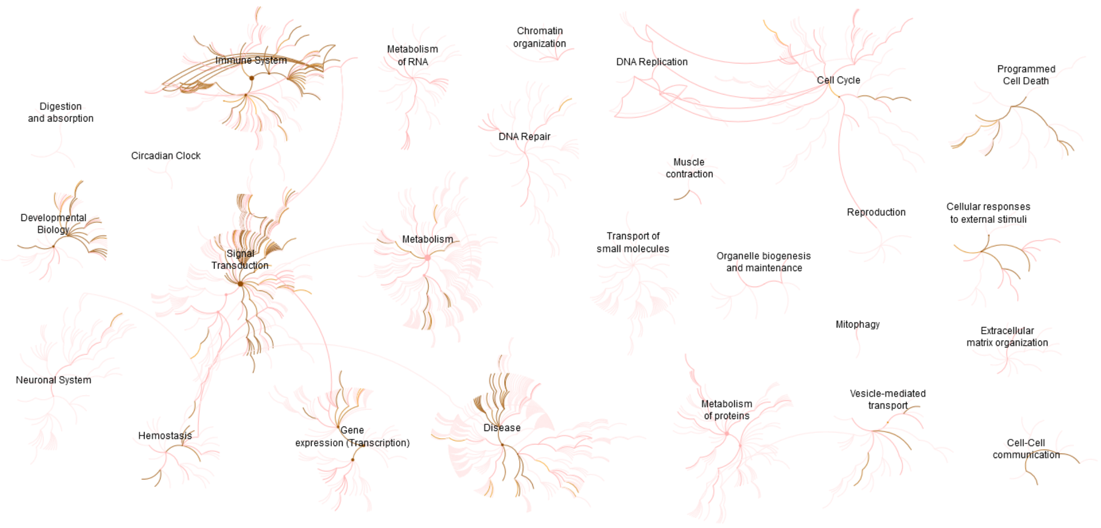

Supplementary Figure 2: Pathway enrichment analysis: visualization. All quantified 52 proteins were analysed using RACTOME pathway enrichment analysis tool REACTOME. A visual representation of the results shows a large coverage of many pathways, e.g. programmed cell death, signal transduction and immune system.

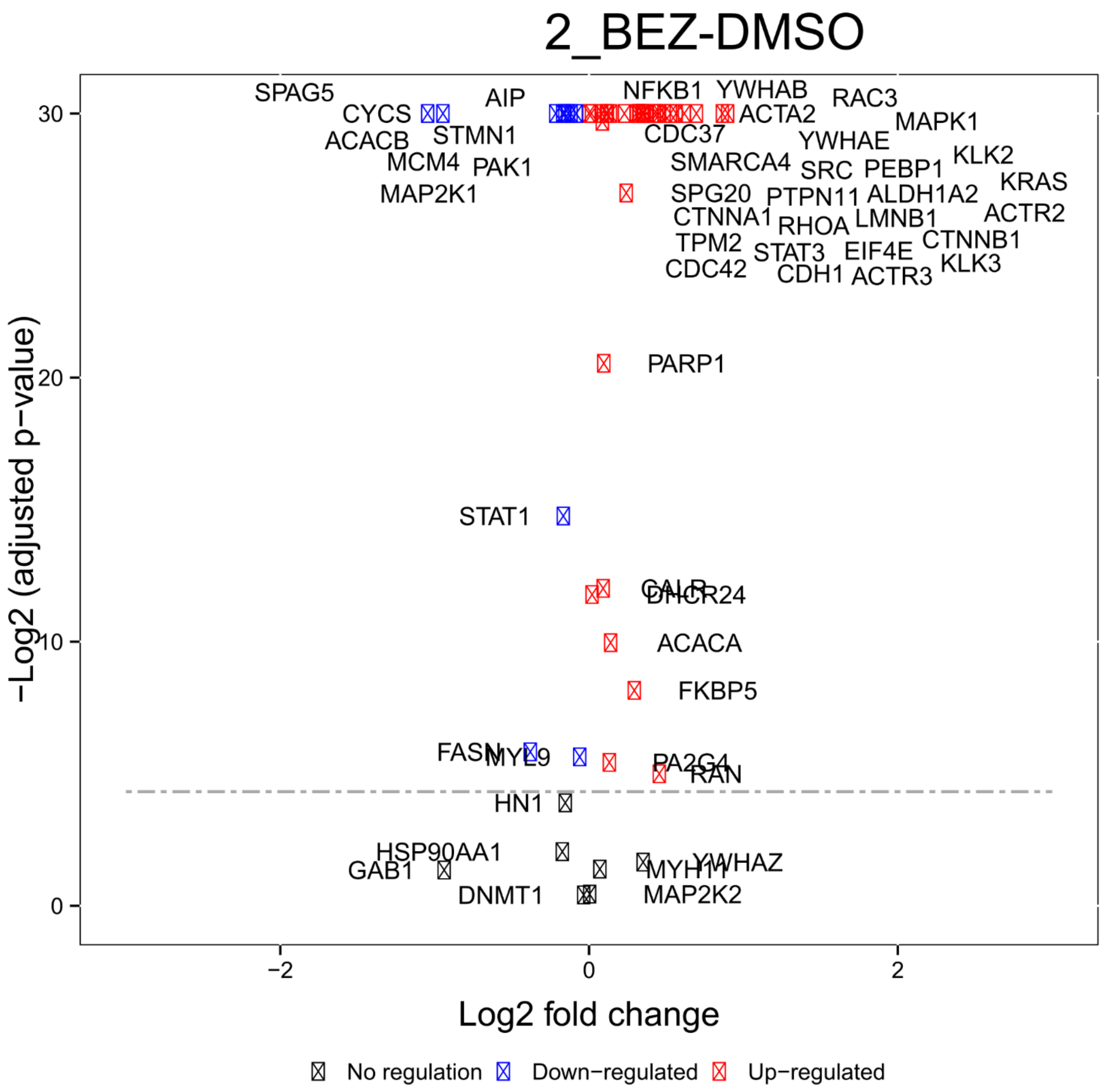
2_BEZ-DMSO
-Log2 (adjusted p-value)
Log2 fold change
SPAG5
CYCS
ACACB
MCM4
MAP2K1
AIP
STMN1
PAK1
NFKB1
YWHAB
RAC3
ACTA2
MAPK1
CDC37
YWHAE
KLK2
SMARCA4
SRC
PEBP1
KRAS
SPG20
PTPN11
ALDH1A2
ACTR2
CTNNA1
RHOA
LMNB1
TPM2
STAT3
EIF4E
CTNNB1
CDC42
CDH1
ACTR3
KLK3
PARP1
STAT1
CALR
DHCR24
ACACA
FKBP5
FASN
MYL9
PA2G4
RAN
HN1
HSP90AA1
GAB1
MYH11
YWHAZ
DNMT1
MAP2K2
30
20
10
0
−2
0
2
No regulation
Down−regulated
Up−regulated

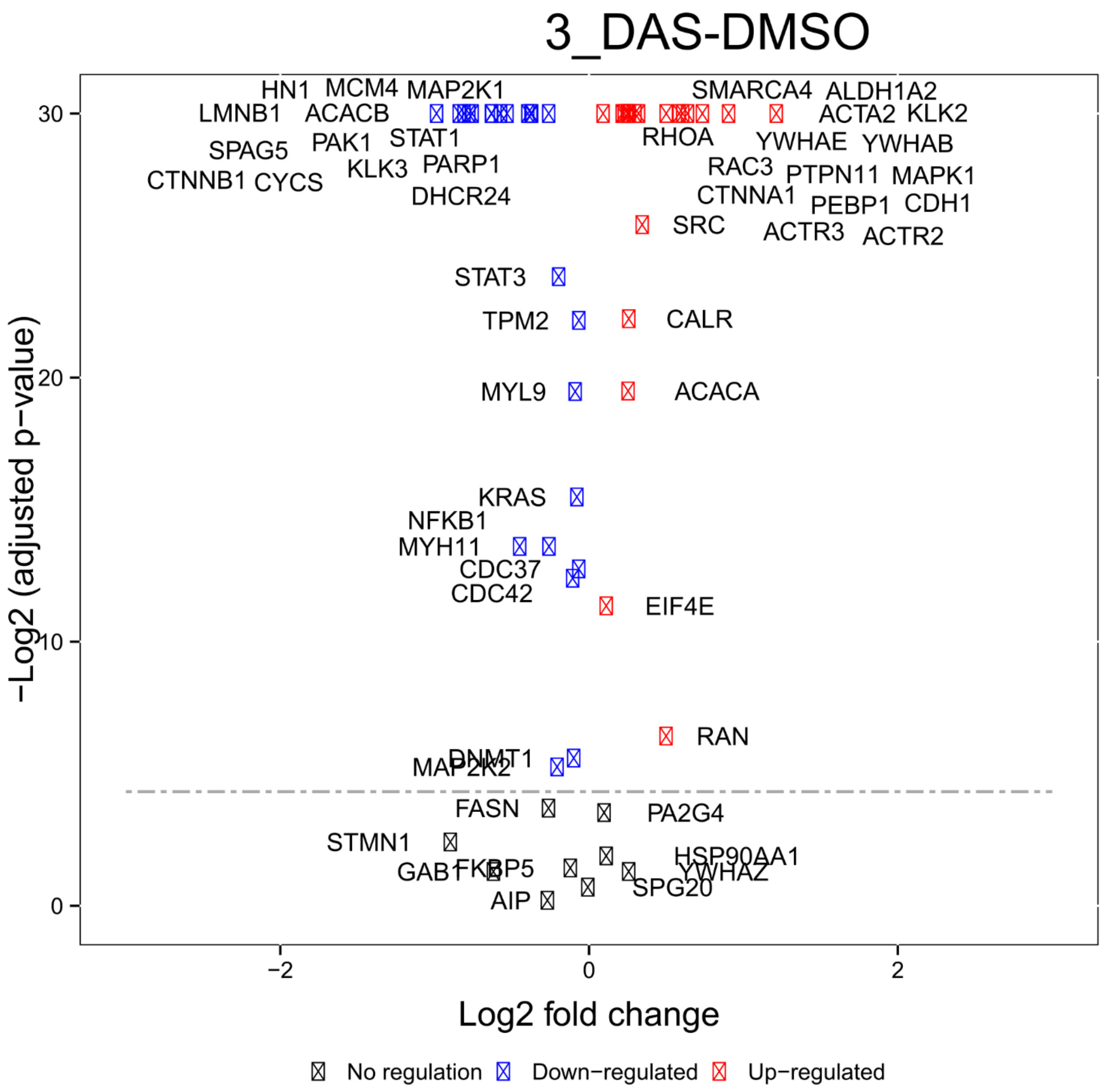
3_DAS-DMSO
-Log2 (adjusted p-value)
Log2 fold change
HN1
MCM4
MAP2K1
LMNB1
ACACB
SPAG5
PAK1
STAT1
CTNNB1
CYCS
KLK3
PARP1
DHCR24
SMARCA4
ALDH1A2
ACTA2
KLK2
RHOA
YWHAE
YWHAB
RAC3
PTPN11
MAPK1
CTNNA1
PEBP1
CDH1
SRC
ACTR3
ACTR2
STAT3
TPM2
CALR
MYL9
ACACA
KRAS
NFKB1
MYH11
CDC37
CDC42
EIF4E
RAN
DNMT1
MAP2K2
FASN
PA2G4
STMN1
HSP90AA1
GAB1
FKBP5
YWHAZ
SPG20
AIP
30
20
10
0
−2
0
2
No regulation
Down−regulated
Up−regulated

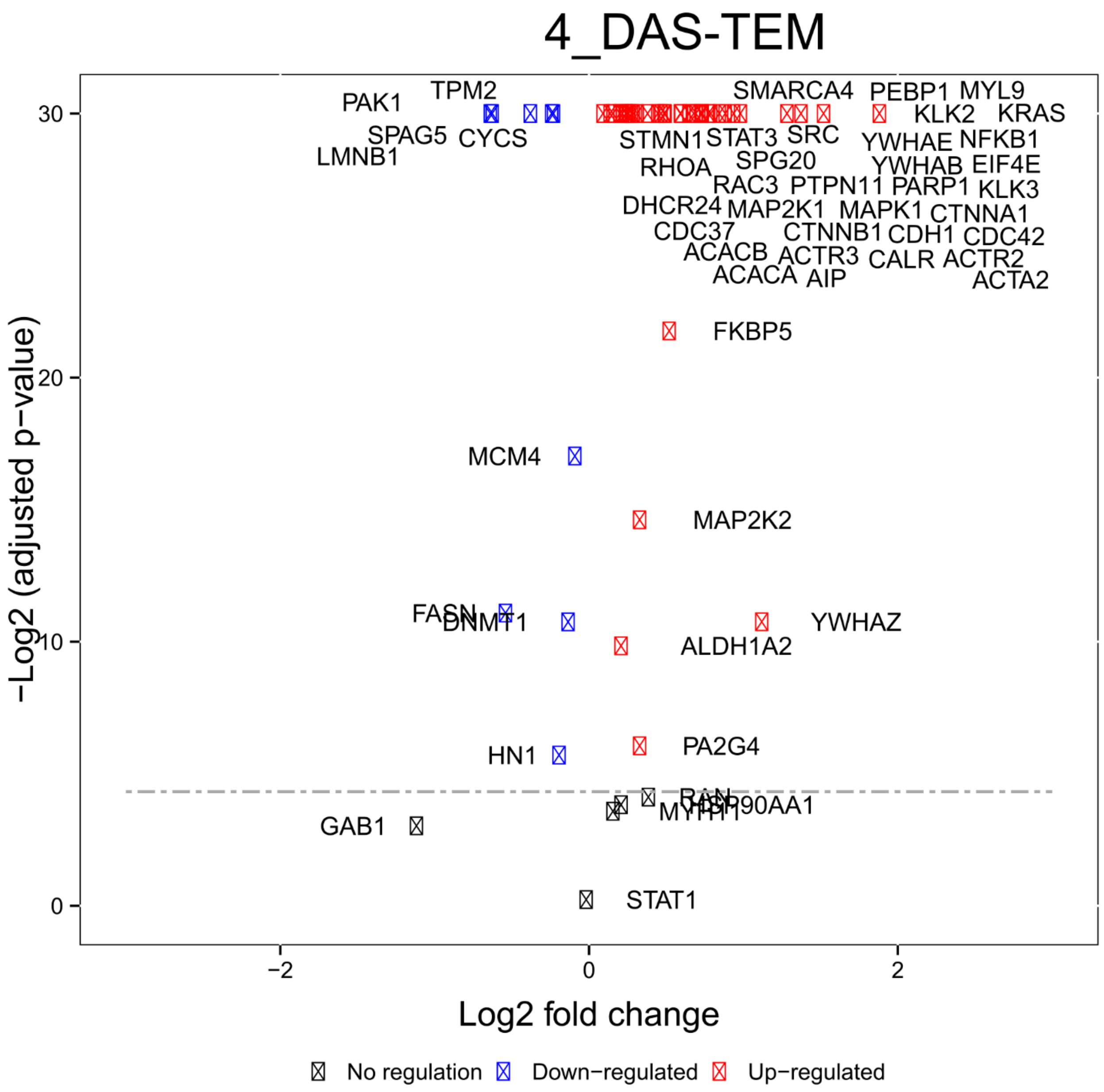
4_DAS-TEM
-Log2 (adjusted p-value)
Log2 fold change
No regulation
Down-regulated
Up-regulated
PAK1
TPM2
SPAG5
CYCS
LMNB1
SMARCA4
PEBP1
MYL9
KLK2
KRAS
STMN1
STAT3
SRC
YWHAE
NFKB1
RHOA
SPG20
YWHAB
EIF4E
RAC3
PTPN11
PARP1
KLK3
DHCR24
MAP2K1
MAPK1
CTNNA1
CDC37
CTNNB1
CDH1
CDC42
ACACB
ACTR3
CALR
ACTR2
ACACA
AIP
ACTA2
FKBP5
MCM4
MAP2K2
YWHAZ
ALDH1A2
PA2G4
HN1
GAB1
STAT1
HSP90AA1
0
10
20
30
−2
0
2

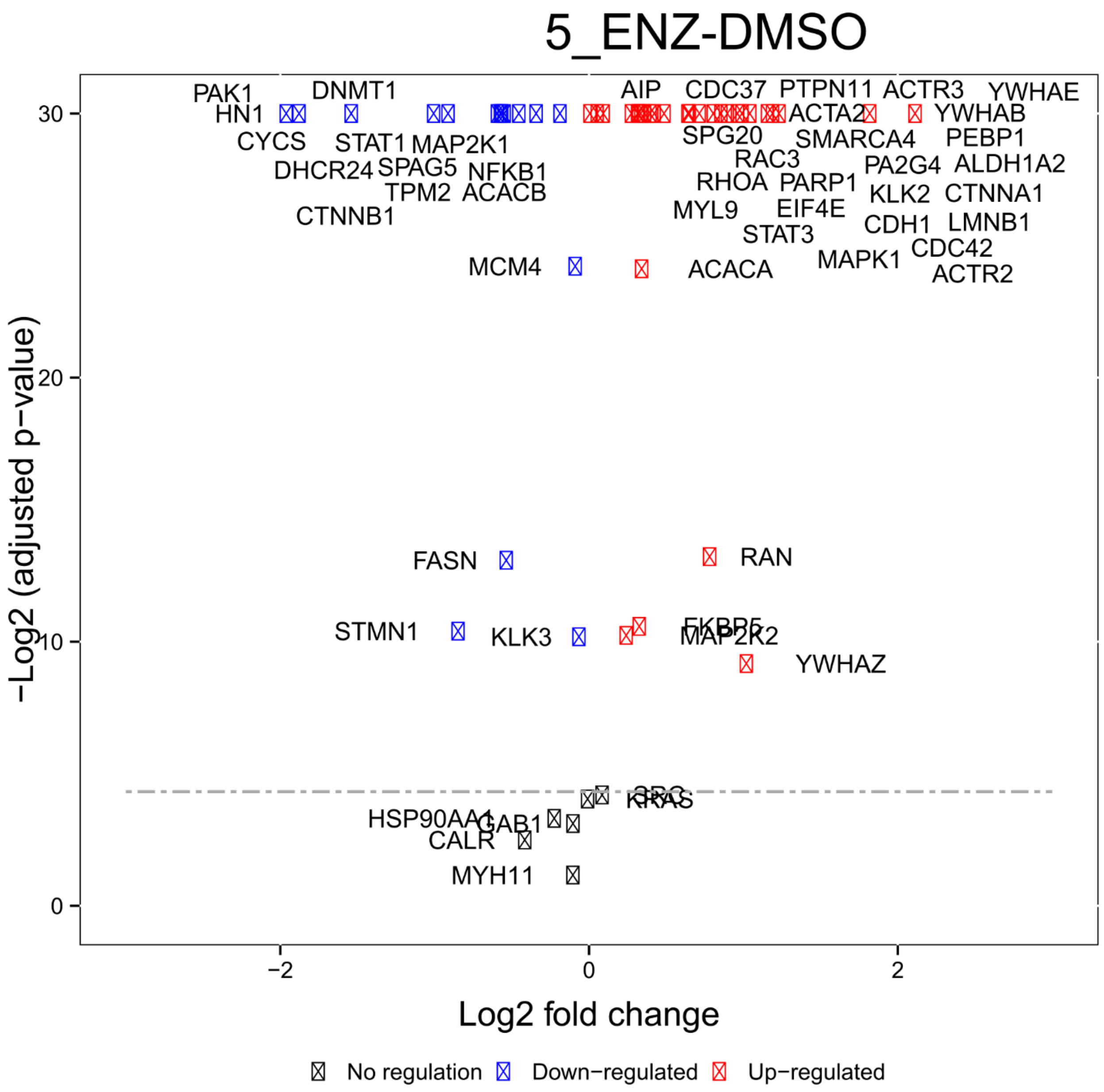
5_ENZ-DMSO
-Log2 (adjusted p-value)
Log2 fold change
PAK1
HN1
DNMT1
CYCS
STAT1
MAP2K1
DHCR24
SPAG5
NFKB1
TPM2
ACACB
CTNNB1
AIP
CDC37
PTPN11
ACTR3
YWHAE
ACTA2
YWHAB
SPG20
SMARCA4
PEBP1
RAC3
PA2G4
ALDH1A2
RHOA
PARP1
KLK2
CTNNA1
MYL9
EIF4E
CDH1
LMNB1
STAT3
CDC42
MAPK1
ACTR2
MCM4
ACACA
FASN
RAN
STMN1
KLK3
MAP2K2
YWHAZ
KRAS
HSP90AA1
GAB1
CALR
MYH11
No regulation
Down−regulated
Up−regulated

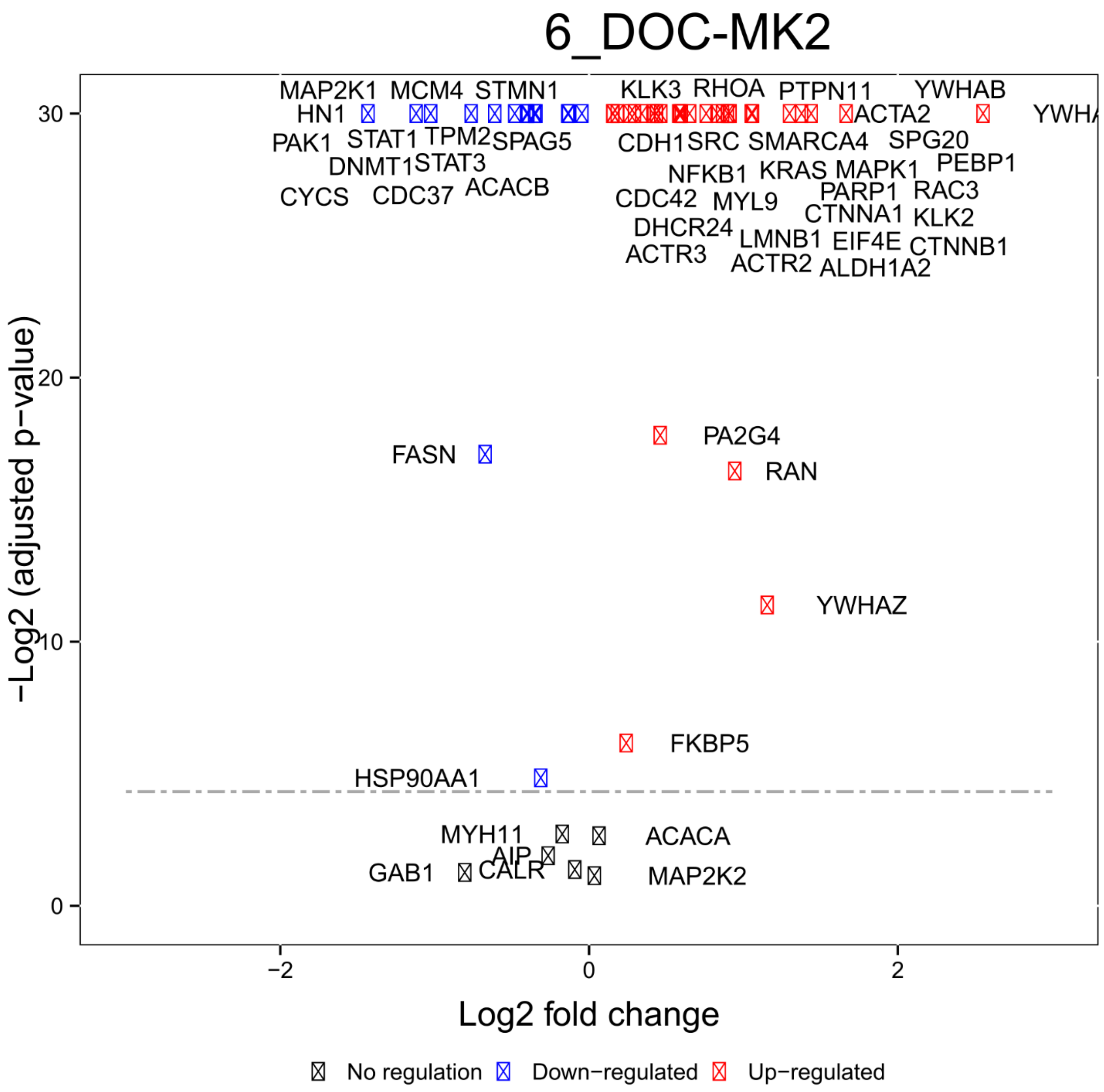
6_DOC-MK2
MAP2K1 MCM4 STMN1
HN1
PAK1 STAT1 TPM2 SPAG5
DNMT1 STAT3
CYCS CDC37 ACACB
KLK3 RHOA PTPN11 YWHAB
ACTA2
YWHA
CDH1 SRC SMARCA4 SPG20
NFKB1 KRAS MAPK1 PEBP1
CDC42 MYL9 PARP1 RAC3
CTNNA1 KLK2
DHCR24 LMNB1 EIF4E CTNNB1
ACTR3 ACTR2 ALDH1A2
FASN
PA2G4
RAN
YWHAZ
FKBP5
HSP90AA1
MYH11
ACACA
AIP
GAB1
CALR
MAP2K2
30
20
10
0
−2
0
2
−Log2 (adjusted p−value)
Log2 fold change
No regulation
Down−regulated
Up−regulated

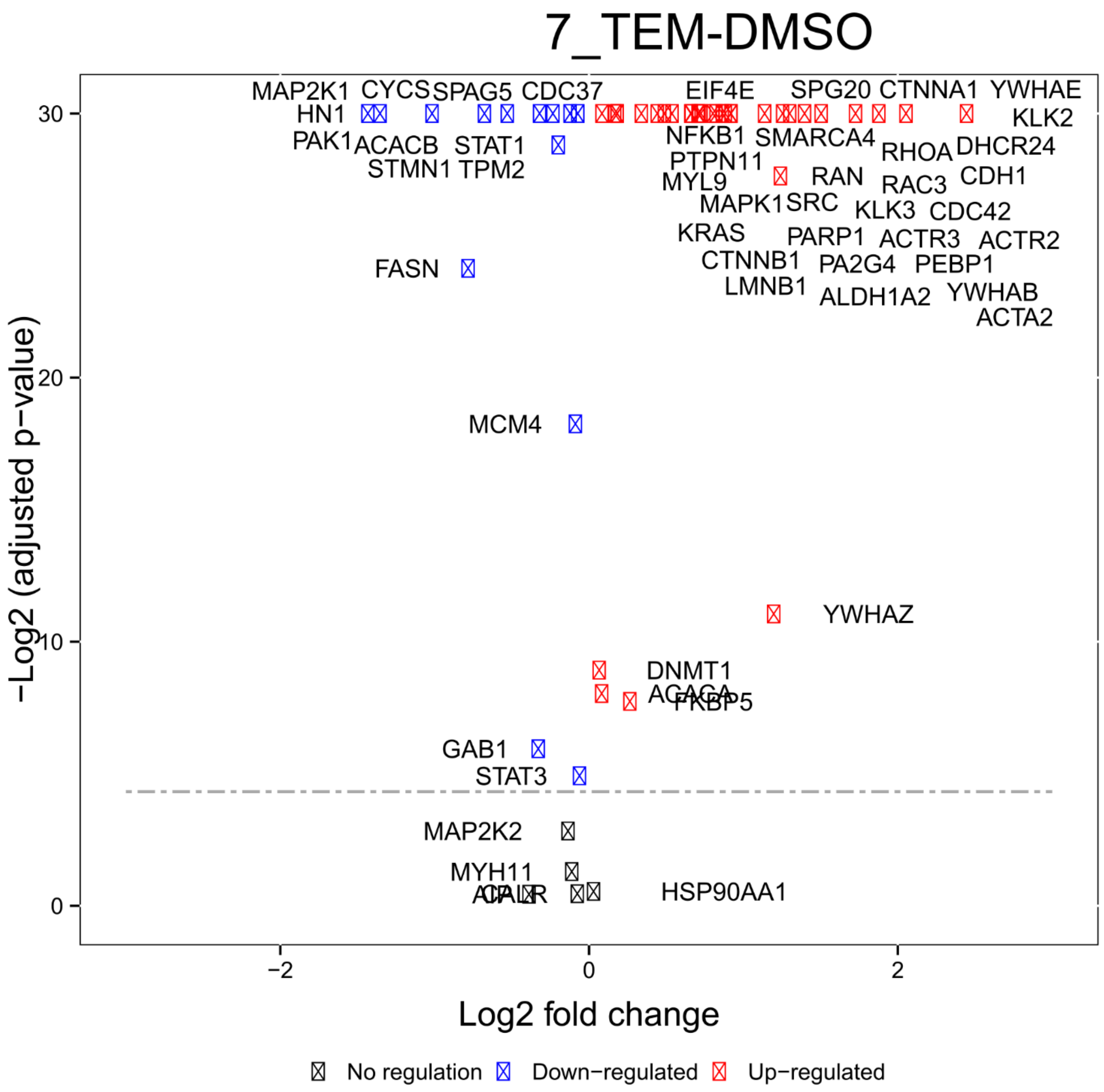
7_TEM-DMSO
MAP2K1
CYCS
SPAG5
CDC37
EIF4E
SPG20
CTNNA1
YWHAE
HN1
KLK2
PAK1
ACACB
STAT1
NFKB1
SMARCA4
RHOA
DHCR24
STMN1
TPM2
PTPN11
MYL9
RAN
RAC3
CDH1
MAPK1
SRC
KLK3
CDC42
KRAS
PARP1
ACTR3
ACTR2
CTNNB1
PA2G4
PEBP1
LMNB1
ALDH1A2
YWHAB
ACTA2
FASN
MCM4
YWHAZ
DNMT1
ACACA
FKBP5
GAB1
STAT3
MAP2K2
MYH11
CALR
ACTA1
HSP90AA1
30
20
10
0
−2
0
2
−Log2 (adjusted p−value)
Log2 fold change
No regulation
Down−regulated
Up−regulated

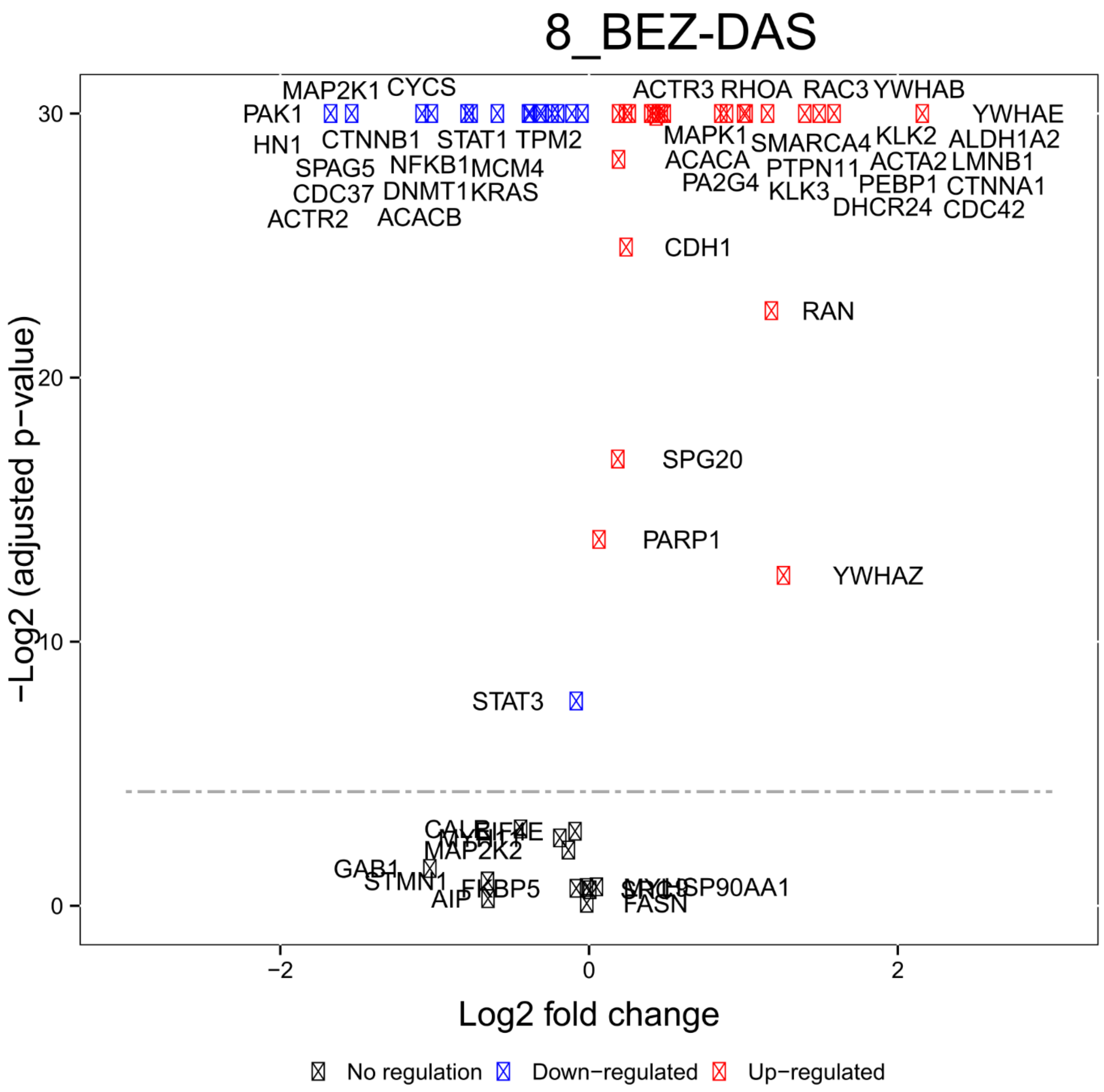
8_BEZ-DAS
−Log2 (adjusted p−value)
30
20
10
0
Log2 fold change
−2
0
2
MAP2K1
CYCS
PAK1
HN1
CTNNB1
STAT1
TPM2
SPAG5
NFKB1
MCM4
CDC37
DNMT1
KRAS
ACTR2
ACACB
ACTR3
RHOA
RAC3
YWHAB
YWHAE
MAPK1
SMARCA4
KLK2
ALDH1A2
ACACA
PTPN11
ACTA2
LMNB1
PA2G4
KLK3
PEBP1
CTNNA1
DHCR24
CDC42
CDH1
RAN
SPG20
PARP1
YWHAZ
STAT3
MAP2K2
GAB1
STMN1
AIP
FKBP5
FASN
No regulation
Down−regulated
Up−regulated

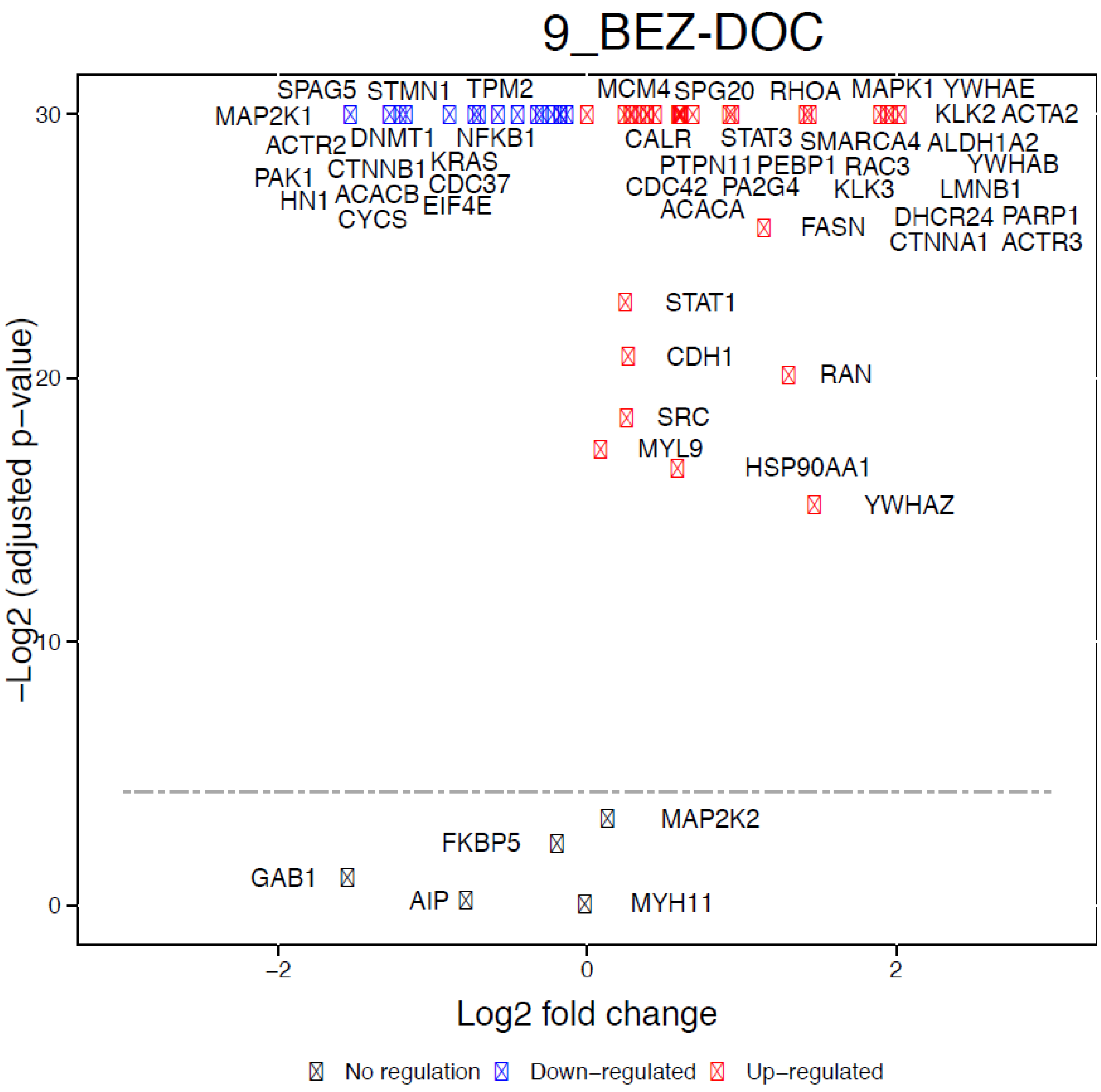
9_BEZ-DOC
SPAG5
STMN1
TPM2
MAP2K1
ACTR2
DNMT1
NFKB1
PAK1
CTNNB1
KRAS
CDC37
HN1
ACACB
EIF4E
CYCS
MCM4
SPG20
RHOA
MAPK1
YWHAE
KLK2
ACTA2
CALR
STAT3
SMARCA4
ALDH1A2
PTPN11
PEBP1
RAC3
YWHAB
CDC42
PA2G4
KLK3
LMNB1
ACACA
FASN
DHCR24
PARP1
CTNNA1
ACTR3
STAT1
CDH1
RAN
SRC
MYL9
HSP90AA1
YWHAZ
MAP2K2
FKBP5
GAB1
AIP
MYH11
30
20
10
0
−2
0
2
−Log2 (adjusted p−value)
Log2 fold change
No regulation
Down−regulated
Up−regulated

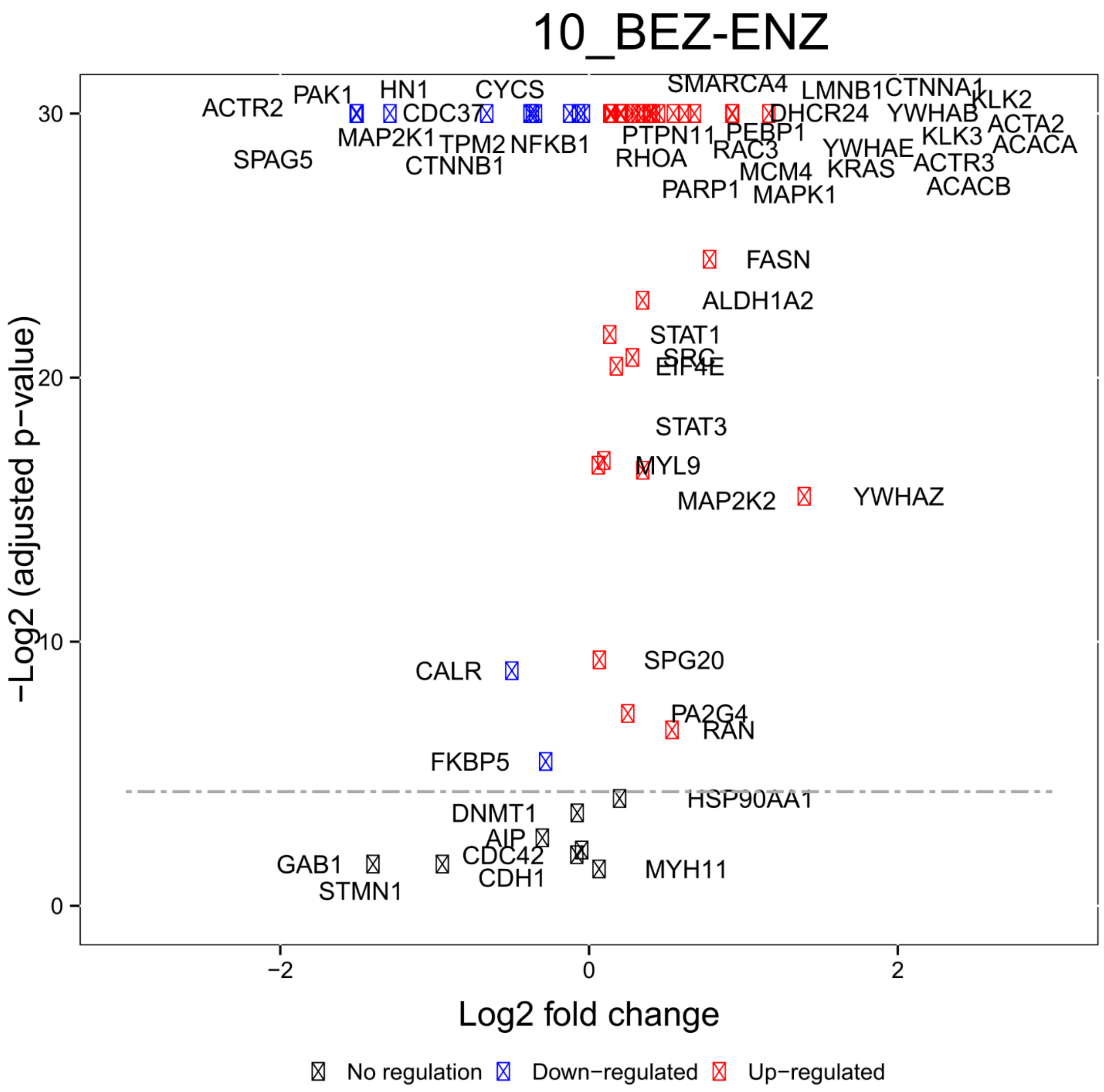
10_BEZ-ENZ
-Log2 (adjusted p-value)
Log2 fold change
30
20
10
0
-2
0
2
ACTR2
PAK1
HN1
CYCS
CDC37
SMARCA4
LMNB1
CTNNA1
KLK2
DHCR24
YWHAB
ACTA2
SPAG5
MAP2K1
TPM2
NFKB1
CTNNB1
PTPN11
PEBP1
RHOA
RAC3
YWHAE
KLK3
ACACA
MCM4
KRAS
ACTR3
PARP1
MAPK1
ACACB
FASN
ALDH1A2
STAT1
SRC
EIF4E
STAT3
MYL9
MAP2K2
YWHAZ
CALR
SPG20
PA2G4
RAN
FKBP5
HSP90AA1
DNMT1
AIP
CDC42
CDH1
GAB1
STMN1
MYH11
No regulation
Down-regulated
Up-regulated

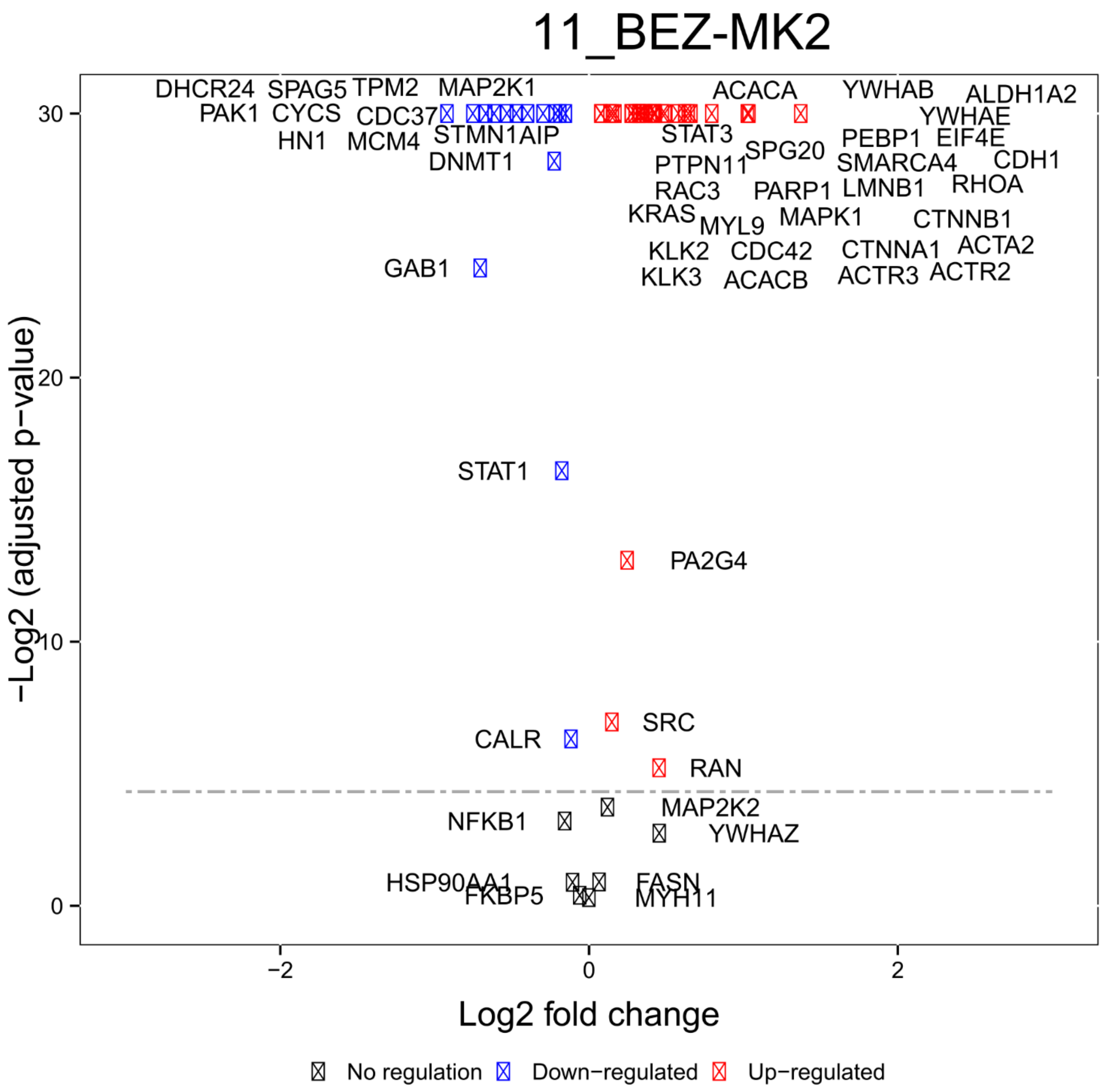
11_BEZ-MK2
-Log2 (adjusted p-value)
Log2 fold change
DHCR24
SPAG5
TPM2
MAP2K1
PAK1
CYCS
CDC37
HN1
MCM4
STMN1
AIP
DNMT1
GAB1
STAT1
CALR
NFKB1
HSP90AA1
FKBP5
ACACA
YWHAB
ALDH1A2
YWHAE
STAT3
PEBP1
EIF4E
SPG20
PTPN11
SMARCA4
CDH1
RAC3
PARP1
LMNB1
RHOA
KRAS
MYL9
MAPK1
CTNNB1
KLK2
CDC42
CTNNA1
ACTA2
KLK3
ACACB
ACTR3
ACTR2
PA2G4
SRC
RAN
MAP2K2
YWHAZ
FASN
MYH11
No regulation
Down−regulated
Up−regulated

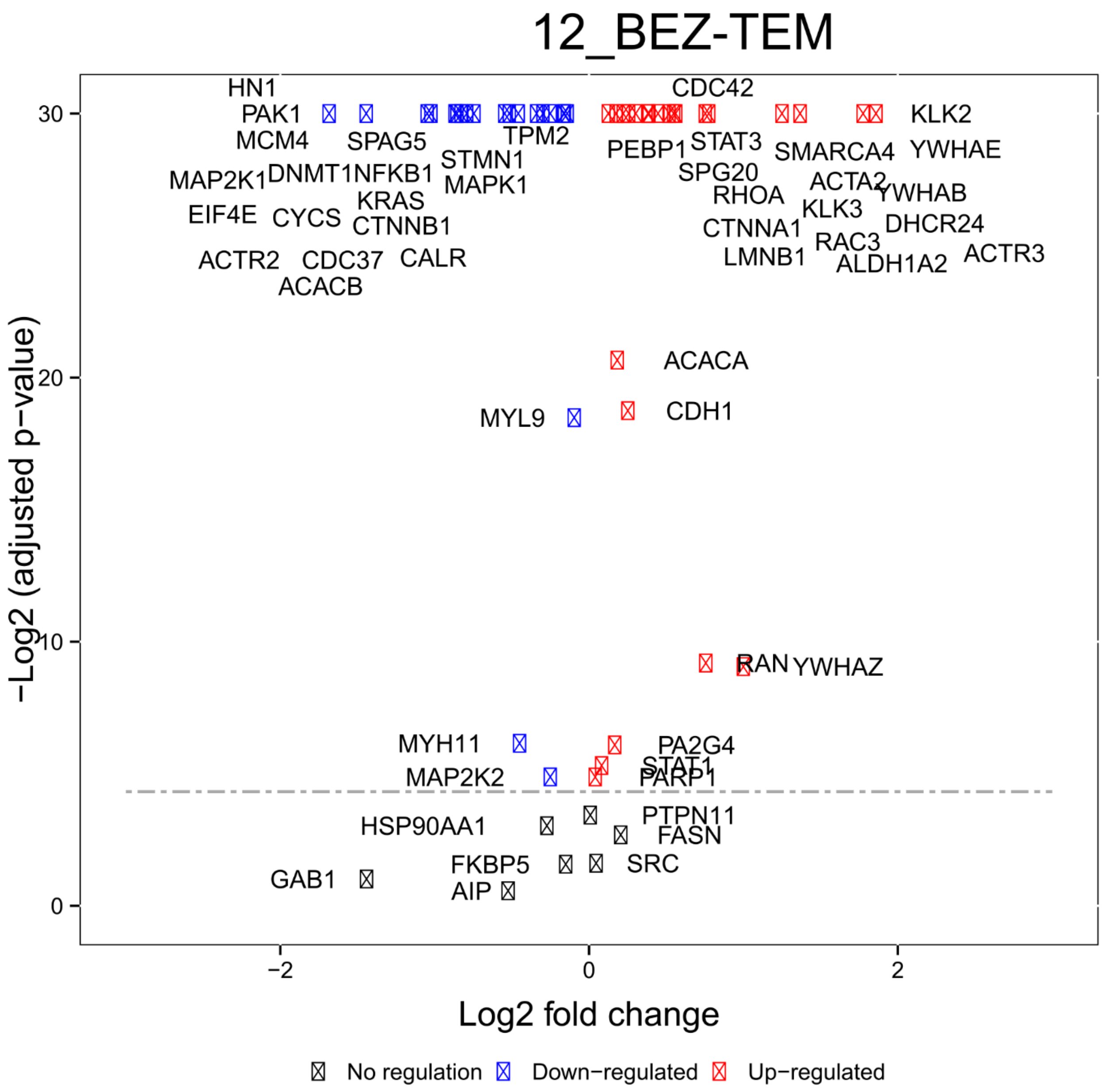
12_BEZ-TEM
−Log2 (adjusted p−value)
Log2 fold change
0
10
20
30
−2
0
2
HN1
PAK1
MCM4
SPAG5
TPM2
STMN1
MAPK1
MAP2K1
DNMT1
NFKB1
KRAS
EIF4E
CYCS
CTNNB1
ACTR2
CDC37
CALR
ACACB
CDC42
KLK2
PEBP1
STAT3
SMARCA4
YWHAE
SPG20
ACTA2
RHOA
YWHAB
KLK3
CTNNA1
DHCR24
RAC3
LMNB1
ALDH1A2
ACTR3
ACACA
MYL9
CDH1
RAN
YWHAZ
MYH11
PA2G4
STAT1
MAP2K2
PARP1
HSP90AA1
PTPN11
FASN
FKBP5
SRC
GAB1
AIP
No regulation
Down−regulated
Up−regulated

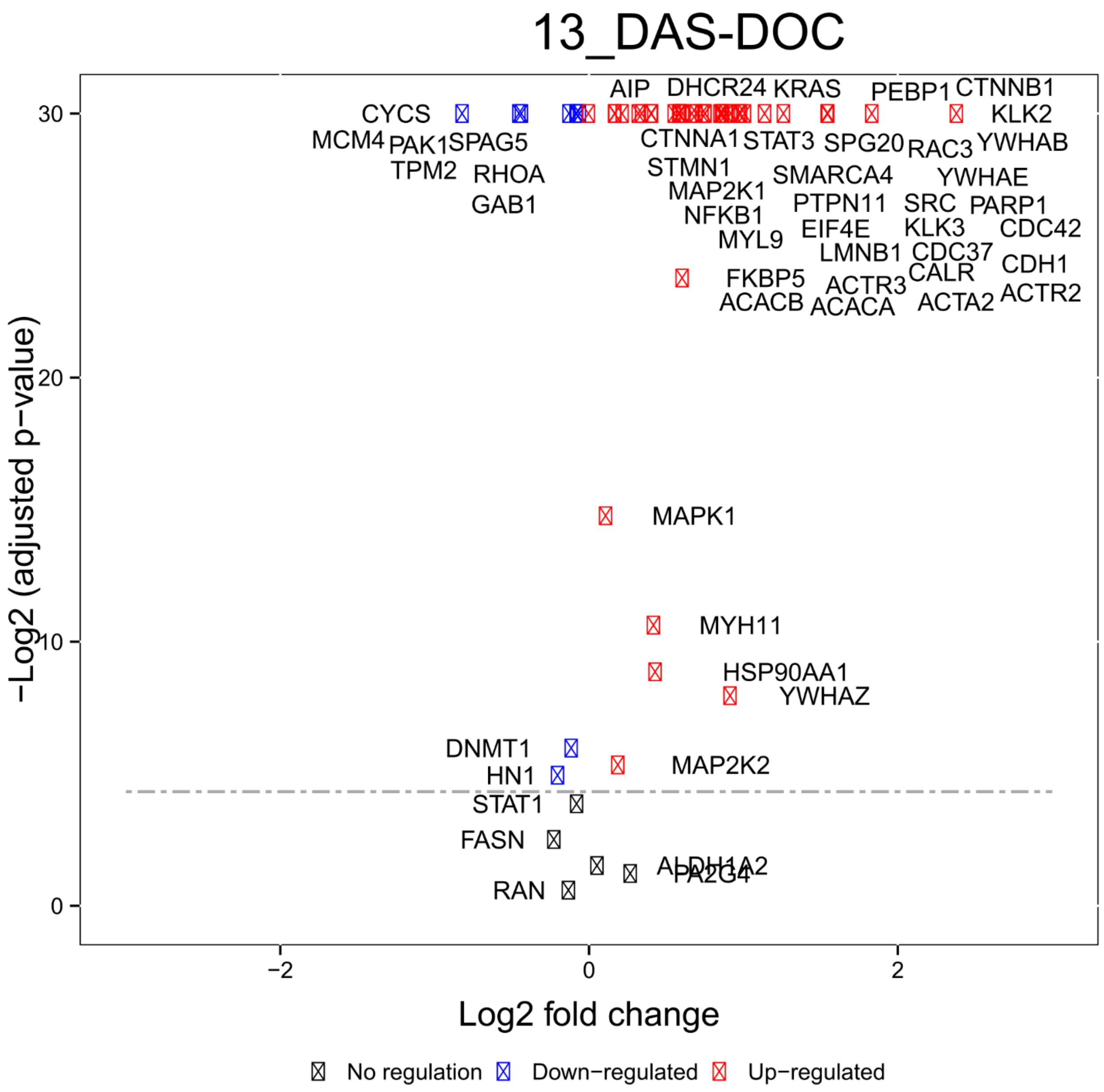
13_DAS-DOC
-Log2 (adjusted p-value)
Log2 fold change
No regulation
Down-regulated
Up-regulated
CYCS
MCM4
PAK1
SPAG5
TPM2
RHOA
GAB1
AIP
DHCR24
KRAS
PEBP1
CTNNB1
KLK2
CTNNA1
STAT3
SPG20
RAC3
YWHAB
STMN1
SMARCA4
YWHAE
MAP2K1
PTPN11
SRC
PARP1
NFKB1
EIF4E
KLK3
CDC42
MYL9
LMNB1
CDC37
CDH1
CALR
FKBP5
ACTR3
ACTR2
ACACB
ACACA
ACTA2
MAPK1
MYH11
HSP90AA1
YWHAZ
DNMT1
MAP2K2
HN1
STAT1
FASN
PA2G4
RAN

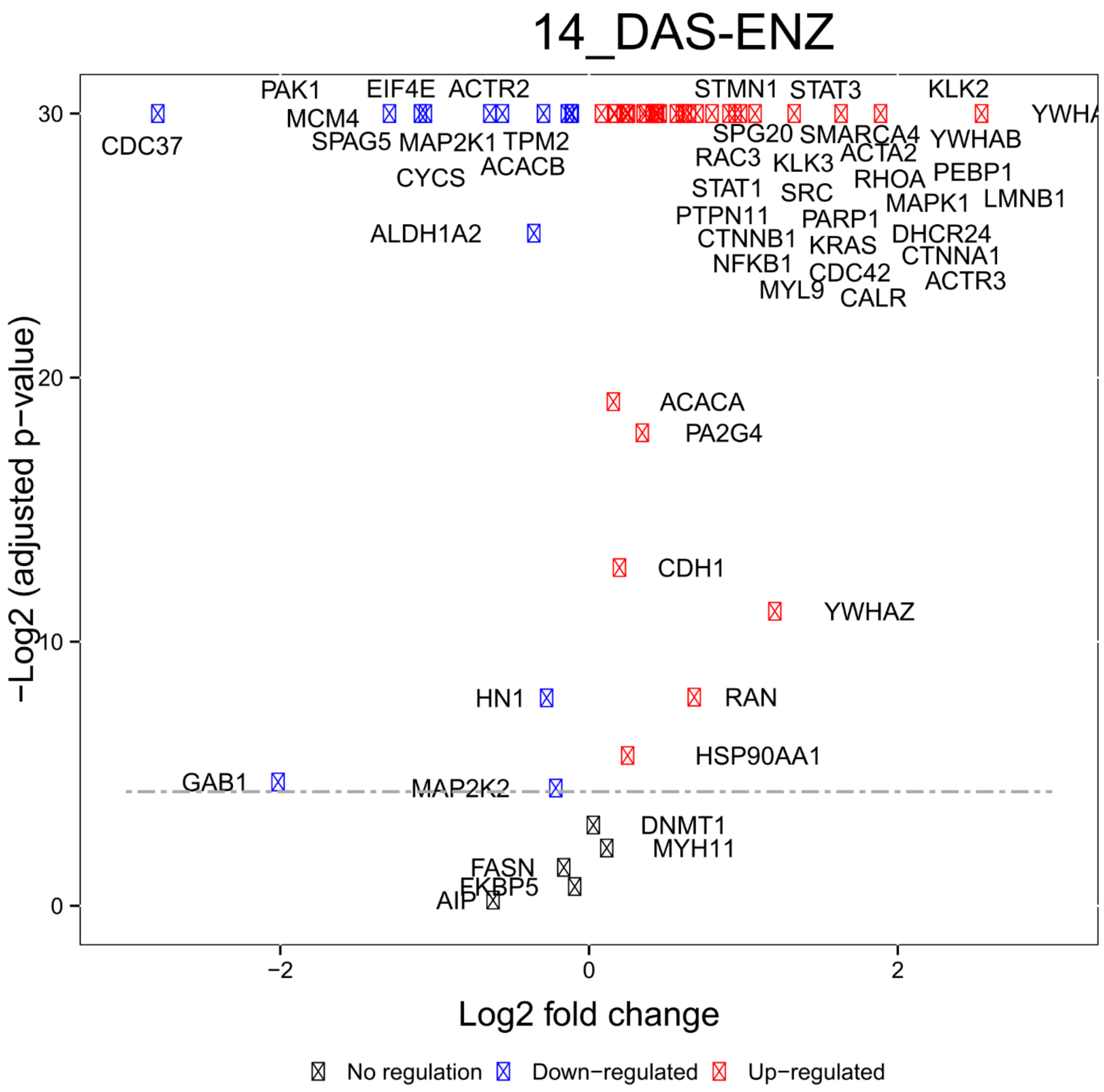
14_DAS-ENZ
-Log2 (adjusted p-value)
Log2 fold change
CDC37
PAK1
MCM4
EIF4E
ACTR2
SPAG5
MAP2K1
TPM2
CYCS
ACACB
ALDH1A2
STMN1
STAT3
KLK2
SPG20
SMARCA4
YWHAB
RAC3
KLK3
ACTA2
RHOA
PEBP1
STAT1
SRC
MAPK1
LMNB1
PTPN11
PARP1
CTNNB1
KRAS
DHCR24
NFKB1
CDC42
CTNNA1
MYL9
CALR
ACTR3
ACACA
PA2G4
CDH1
YWHAZ
HN1
RAN
HSP90AA1
GAB1
MAP2K2
DNMT1
MYH11
FASN
FKBP5
AIP
No regulation
Down−regulated
Up−regulated

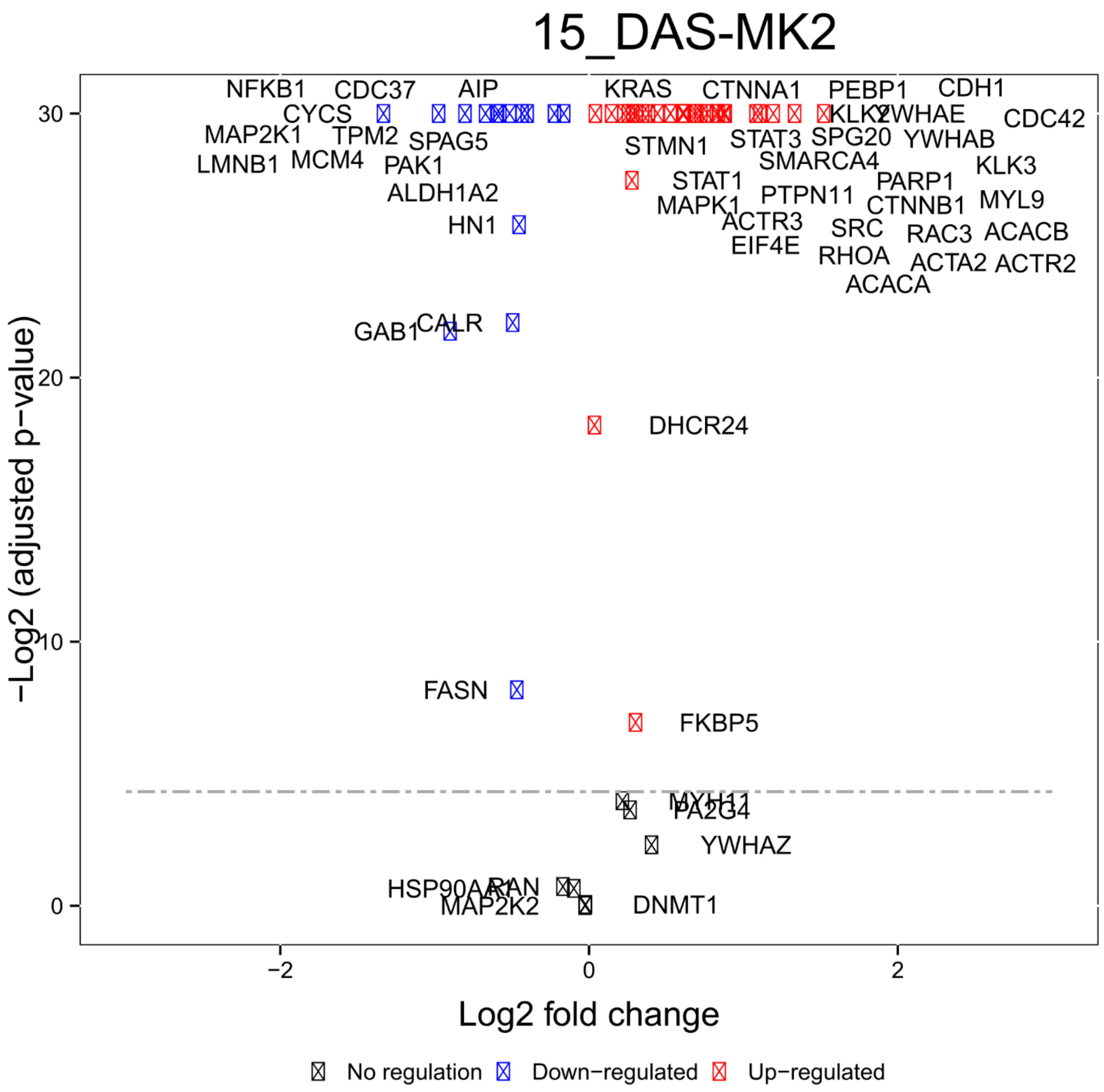
15_DAS-MK2
−Log2 (adjusted p−value)
Log2 fold change
No regulation
Down−regulated
Up−regulated
NFKB1
CDC37
AIP
CYCS
MAP2K1
TPM2
SPAG5
LMNB1
MCM4
PAK1
ALDH1A2
HN1
GAB1
CALR
FASN
KRAS
CTNNA1
PEBP1
CDH1
YWHAE
CDC42
STMN1
STAT3
SPG20
YWHAB
SMARCA4
KLK3
STAT1
PARP1
MAPK1
PTPN11
CTNNB1
MYL9
ACTR3
SRC
RAC3
ACACB
EIF4E
RHOA
ACTA2
ACTR2
ACACA
DHCR24
FKBP5
PA2G4
YWHAZ
DNMT1
MAP2K2
30
20
10
0
−2
0
2

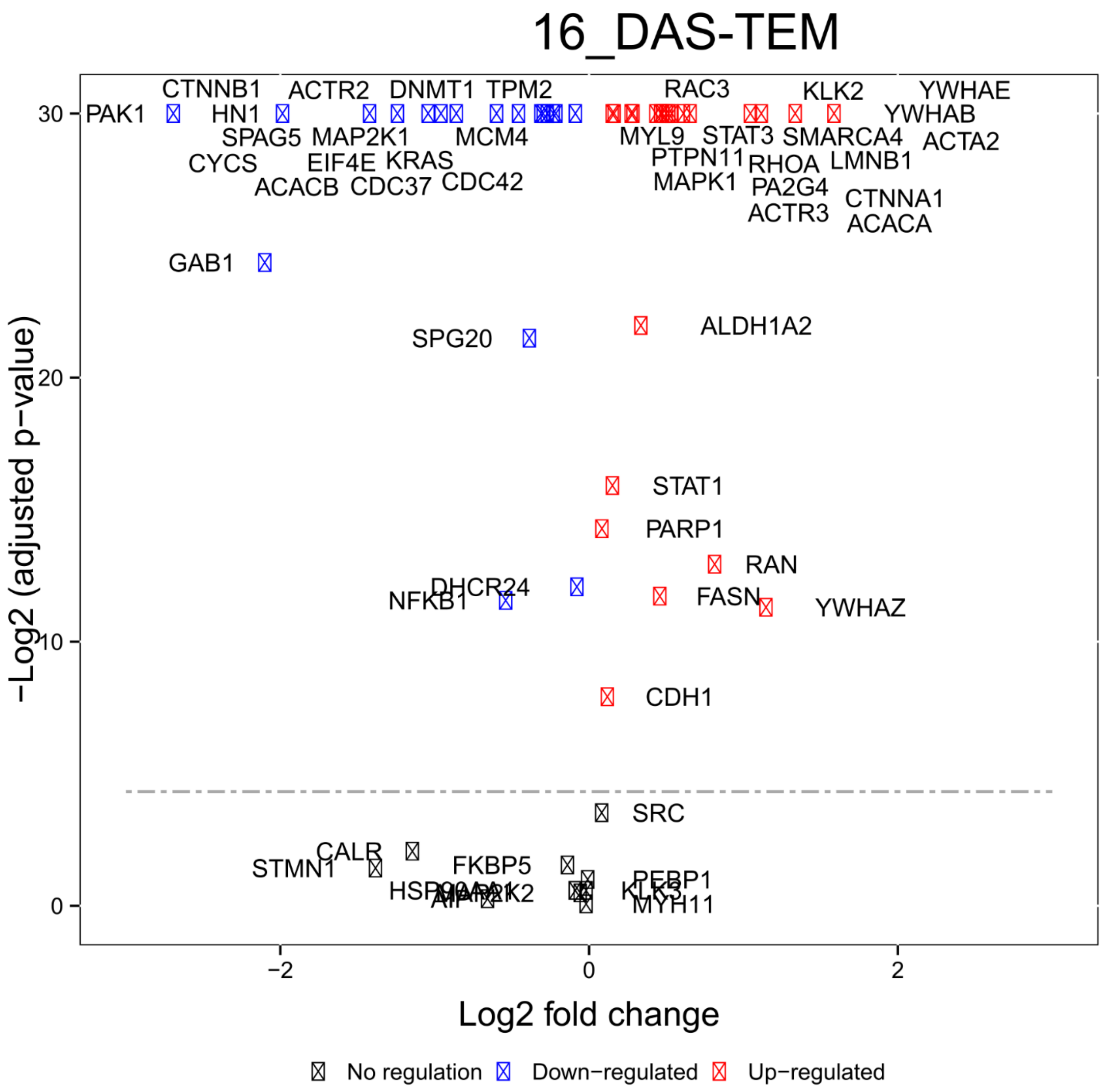
16_DAS-TEM
CTNNB1
ACTR2
DNMT1
TPM2
RAC3
KLK2
YWHAE
PAK1
HN1
YWHAB
SPAG5
MAP2K1
MCM4
MYL9
STAT3
SMARCA4
ACTA2
CYCS
EIF4E
KRAS
PTPN11
RHOA
LMNB1
ACACB
CDC37
CDC42
MAPK1
PA2G4
CTNNA1
ACTR3
ACACA
GAB1
SPG20
ALDH1A2
STAT1
PARP1
RAN
DHCR24
NFKB1
FASN
YWHAZ
CDH1
SRC
CALR
STMN1
FKBP5
PEBP1
KLK3
MYH11
−Log2 (adjusted p−value)
30
20
10
0
−2
0
2
Log2 fold change
No regulation
Down−regulated
Up−regulated

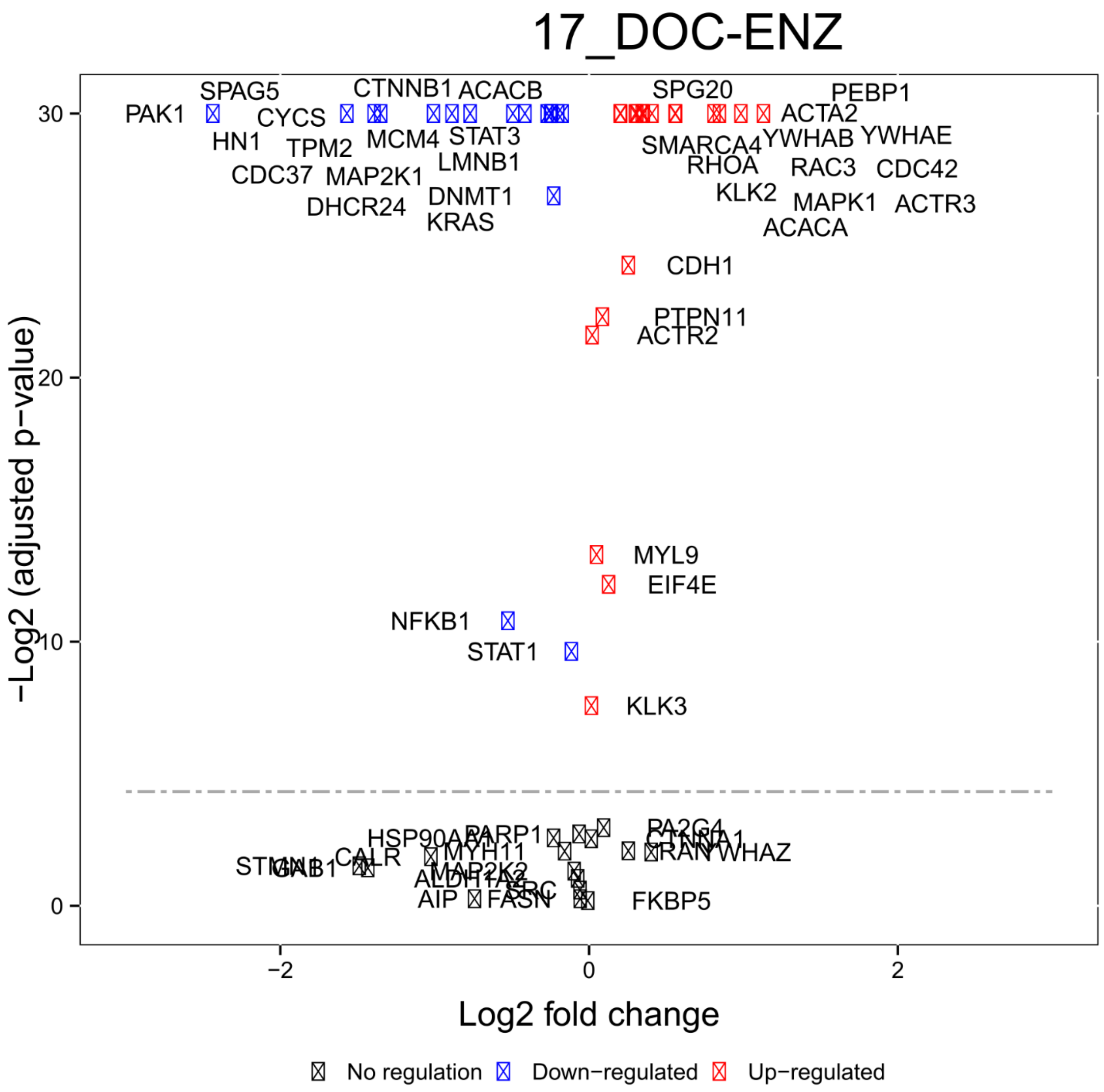
17_DOC-ENZ
−Log2 (adjusted p−value)
Log2 fold change
30
20
10
0
−2
0
2
SPAG5
PAK1
CYCS
HN1
TPM2
CDC37
MAP2K1
DHCR24
CTNNB1
ACACB
MCM4
STAT3
LMNB1
DNMT1
KRAS
SPG20
PEBP1
ACTA2
SMARCA4
YWHAB
YWHAE
RHOA
RAC3
CDC42
KLK2
MAPK1
ACTR3
ACACA
CDH1
PTPN11
ACTR2
MYL9
EIF4E
NFKB1
STAT1
KLK3
CALR
PARP1
MYH11
MAP2K2
SRC
AIP
FASN
FKBP5
PA2G4
RAN
YWHAZ
No regulation
Down−regulated
Up−regulated

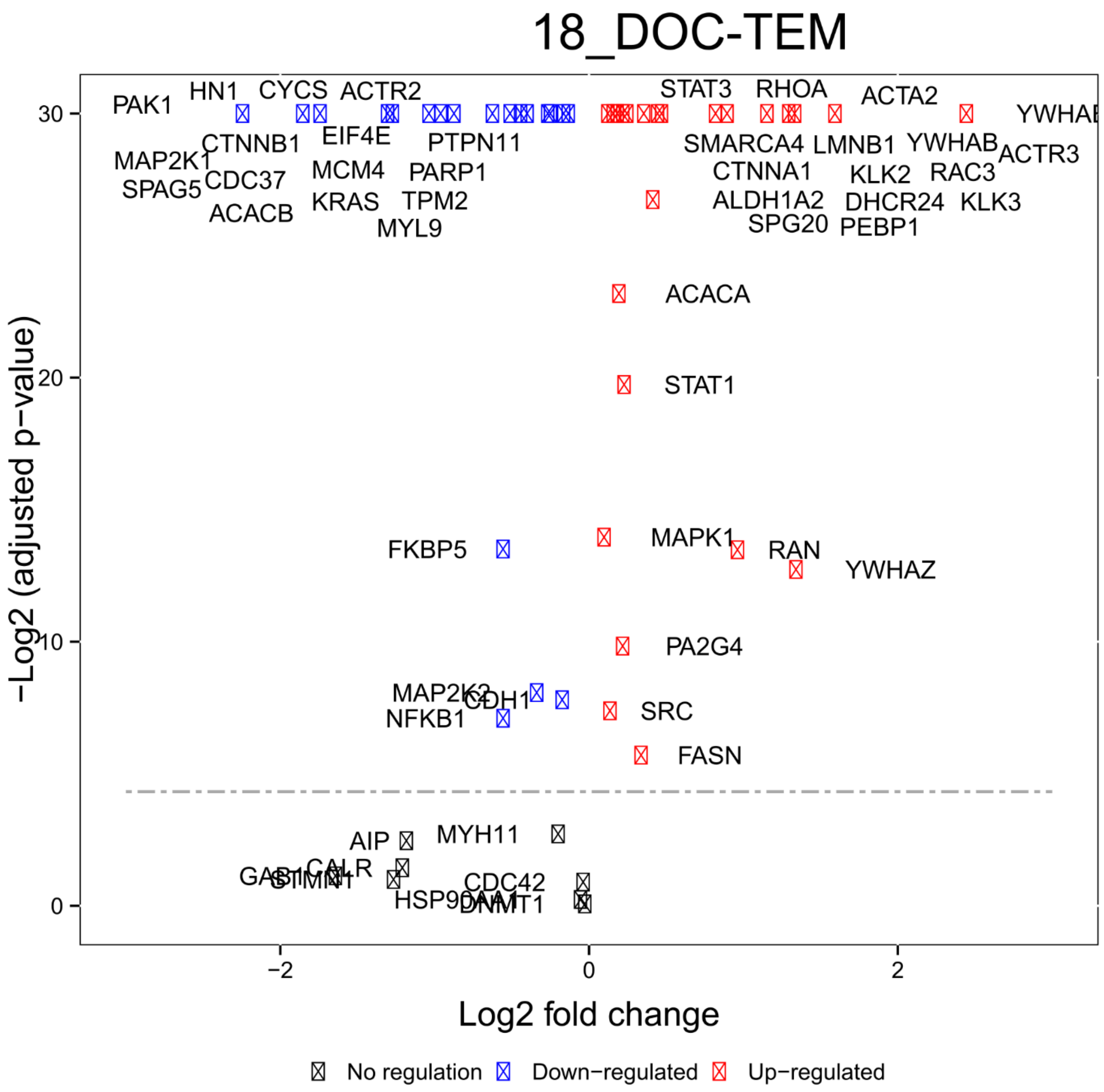
18_DOC-TEM
PAK1
HN1
CYCS
ACTR2
STAT3
RHOA
ACTA2
YWHAE
MAP2K1
CTNNB1
EIF4E
PTPN11
SMARCA4
LMNB1
YWHAB
ACTR3
SPAG5
CDC37
MCM4
PARP1
CTNNA1
KLK2
RAC3
ACACB
KRAS
TPM2
ALDH1A2
DHCR24
KLK3
MYL9
SPG20
PEBP1
ACACA
STAT1
FKBP5
MAPK1
RAN
YWHAZ
PA2G4
SRC
NFKB1
FASN
AIP
MYH11
CDC42
-Log2 (adjusted p-value)
Log2 fold change
No regulation
Down-regulated
Up-regulated

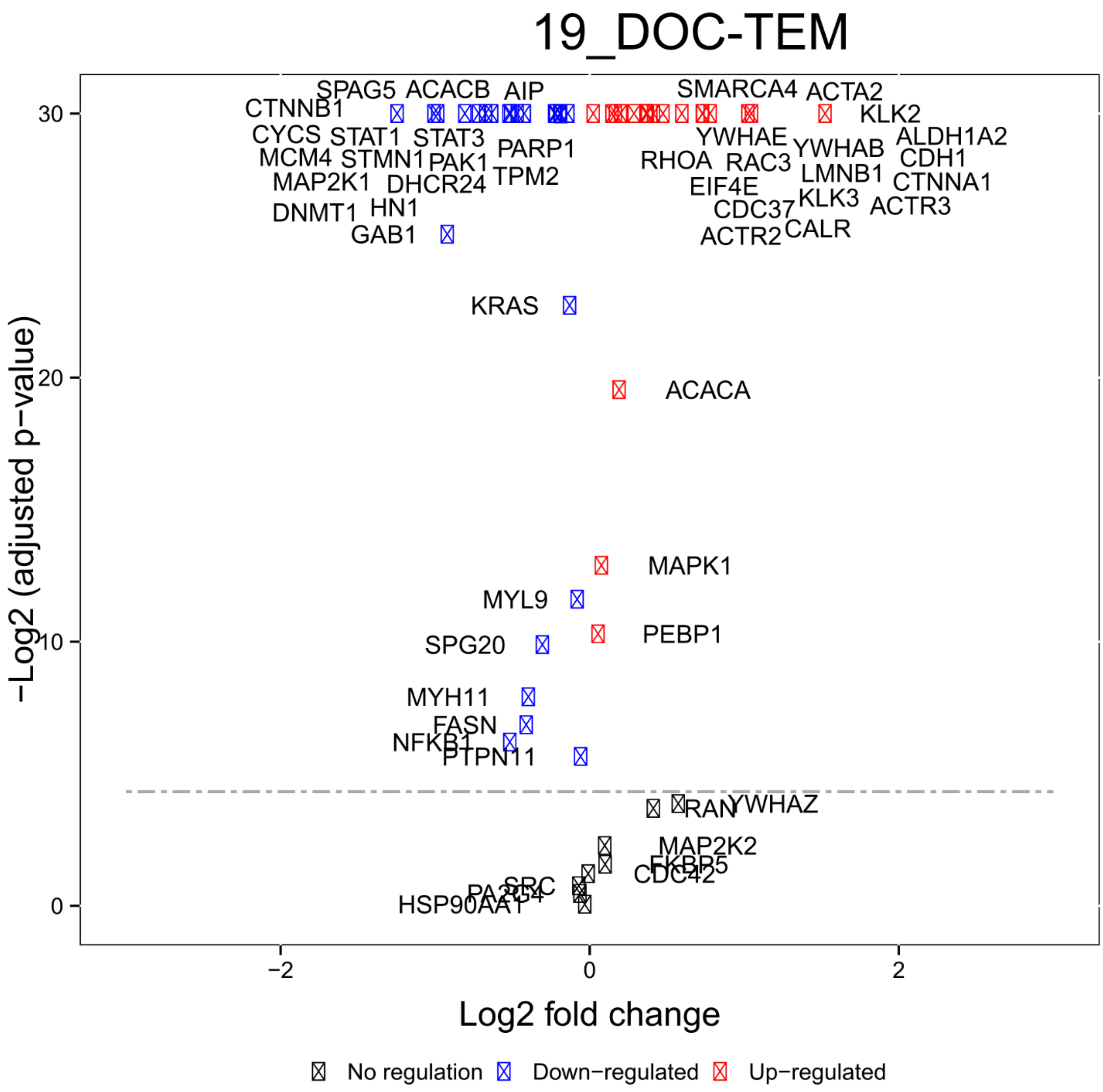
19_DOC-TEM
SPAG5 ACACB AIP
CTNNB1
SMARCA4 ACTA2
KLK2
CYCS STAT1 STAT3
MCM4 STMN1 PAK1 PARP1
MAP2K1 DHCR24 TPM2
DNMT1 HN1
GAB1
YWHAE
RHOA RAC3 YWHAB
ALDH1A2
CDH1
LMNB1
CTNNA1
EIF4E
CDC37 KLK3 ACTR3
ACTR2 CALR
KRAS
ACACA
MAPK1
MYL9
SPG20
PEBP1
MYH11
FASN
NFKB1
PTPN11
RAN YWHAZ
MAP2K2
FKBP5
CDC42
SRC
PA2G4
HSP90AA1
30
20
10
0
−2
0
2
−Log2 (adjusted p−value)
Log2 fold change
No regulation
Down−regulated
Up−regulated

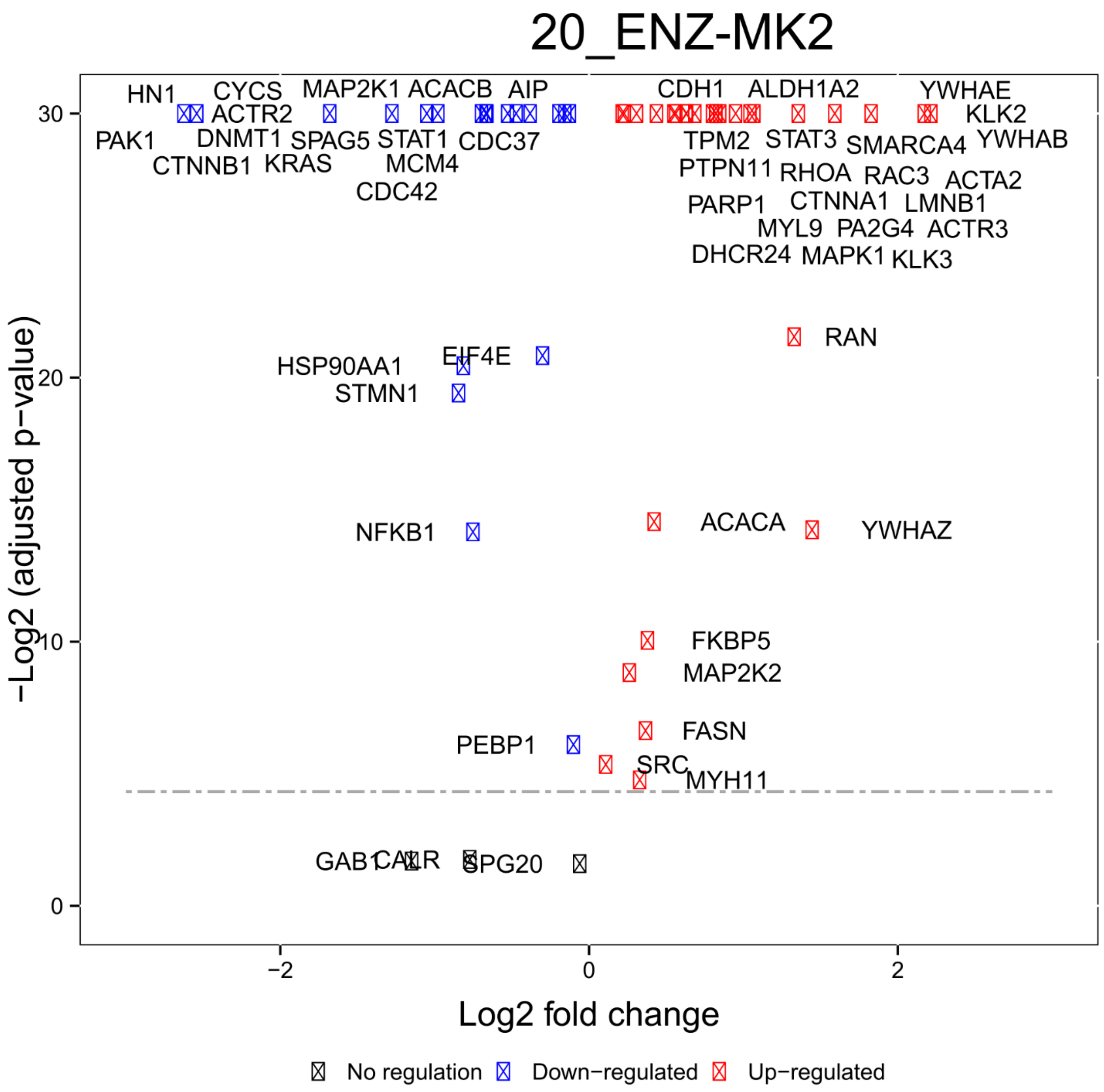
20_ENZ-MK2
HN1
CYCS
ACTR2
MAP2K1
ACACB
AIP
CDH1
ALDH1A2
YWHAE
KLK2
PAK1
DNMT1
SPAG5
STAT1
CDC37
TPM2
STAT3
SMARCA4
YWHAB
CTNNB1
KRAS
MCM4
CDC42
PTPN11
RHOA
RAC3
ACTA2
PARP1
CTNNA1
LMNB1
MYL9
PA2G4
ACTR3
DHCR24
MAPK1
KLK3
RAN
HSP90AA1
EIF4E
STMN1
NFKB1
ACACA
YWHAZ
FKBP5
MAP2K2
FASN
PEBP1
SRC
MYH11
GAB1
CALR
SPG20
30
20
10
0
−2
0
2
−Log2 (adjusted p−value)
Log2 fold change
No regulation
Down−regulated
Up−regulated

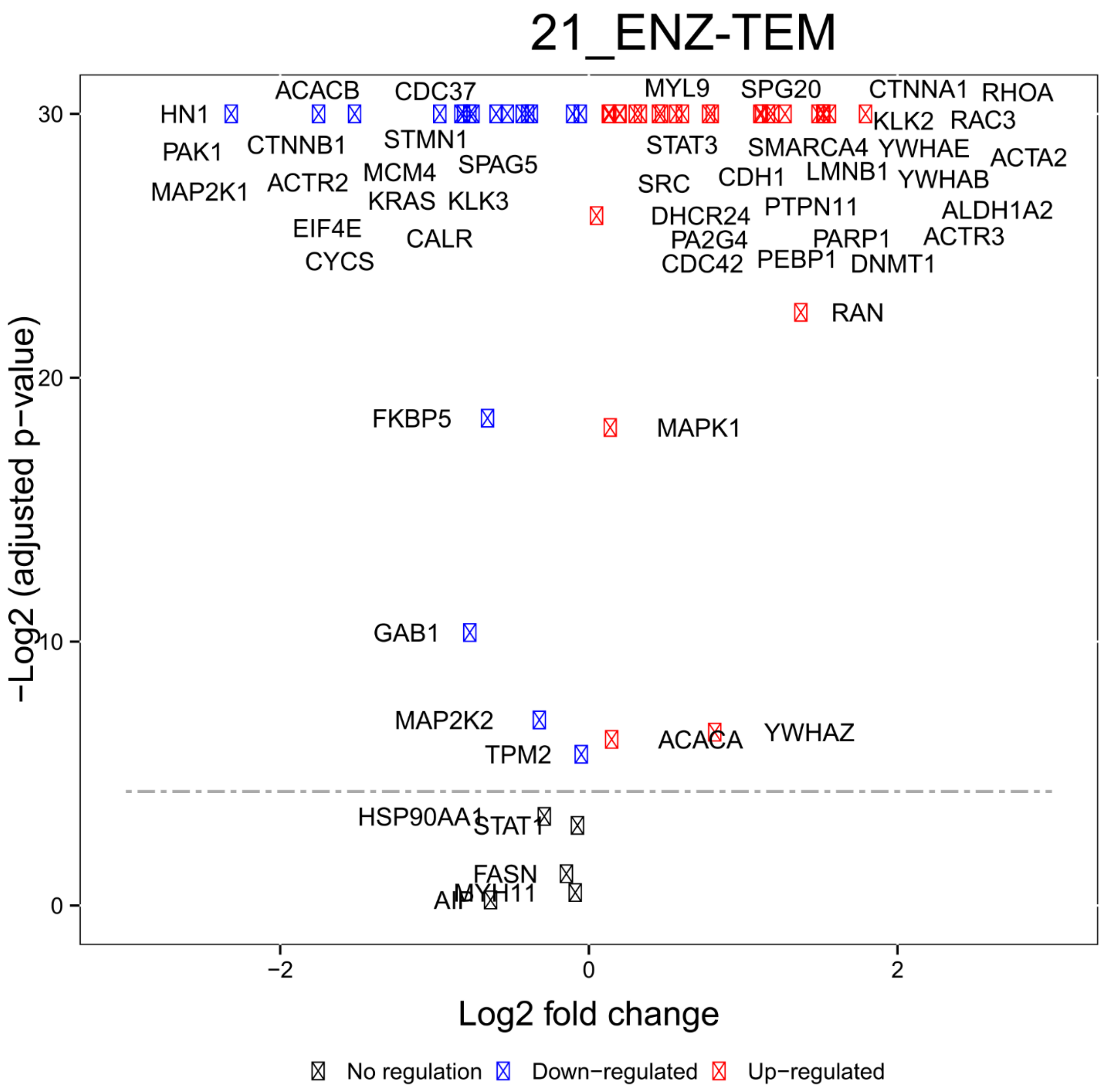
21_ENZ-TEM
-Log2 (adjusted p-value)
Log2 fold change
No regulation
Down-regulated
Up-regulated
HN1
ACACB
CDC37
MYL9
SPG20
CTNNA1
RHOA
KLK2
RAC3
PAK1
CTNNB1
STMN1
STAT3
SMARCA4
YWHAE
ACTA2
MAP2K1
ACTR2
MCM4
SPAG5
SRC
CDH1
LMNB1
YWHAB
KRAS
KLK3
DHCR24
PTPN11
ALDH1A2
EIF4E
CALR
PA2G4
PARP1
ACTR3
CYCS
CDC42
PEBP1
DNMT1
RAN
FKBP5
MAPK1
GAB1
MAP2K2
ACACA
YWHAZ
TPM2
HSP90AA1
STAT1
FASN
AIP
MYH11
30
20
10
0
−2
0
2

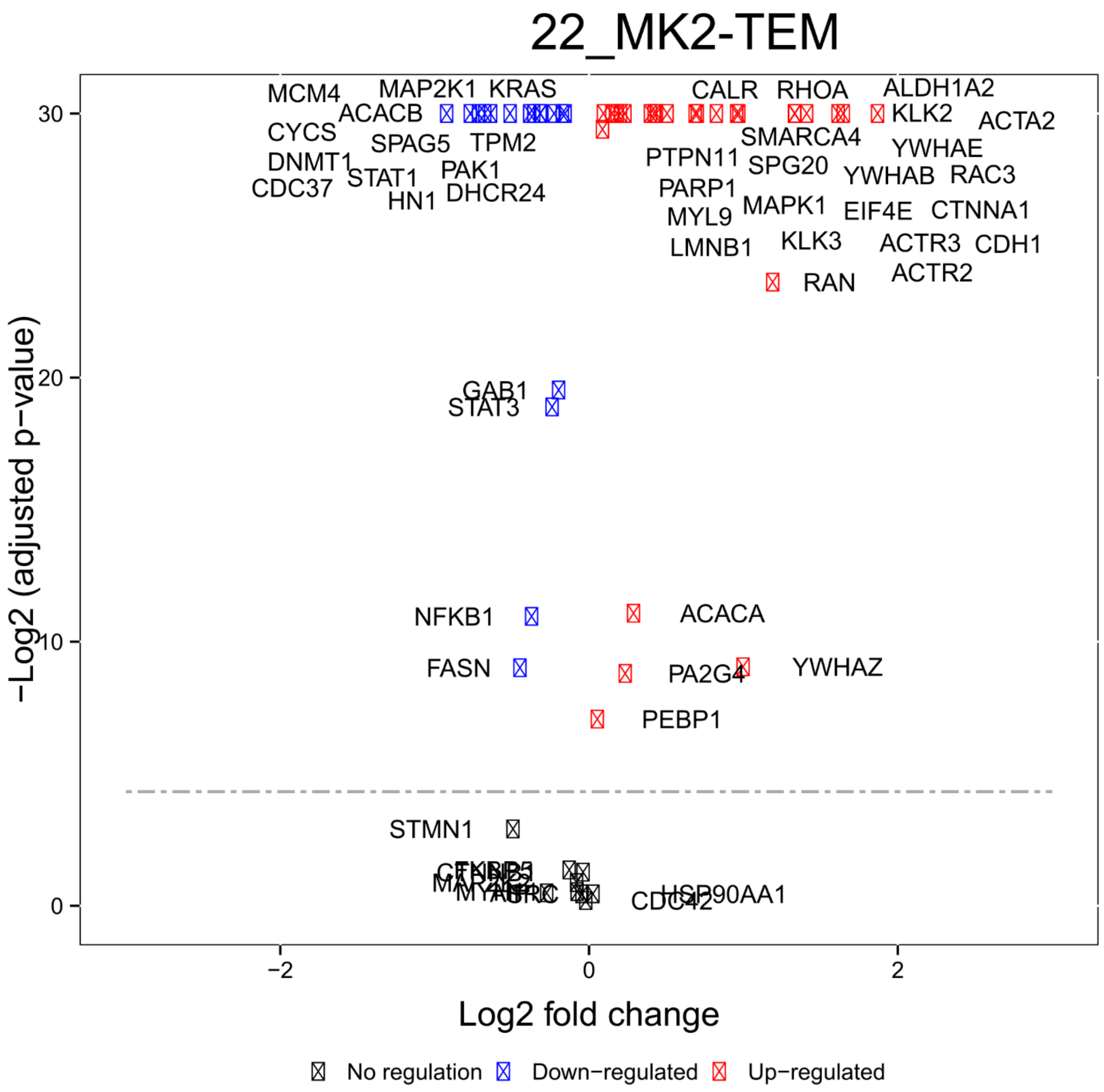

Supplementary Figure 3: SRM data analysis I. 21 primary output of MSStats per condition plotting statistical significance as function of protein abundance ratio between treated and untreated.

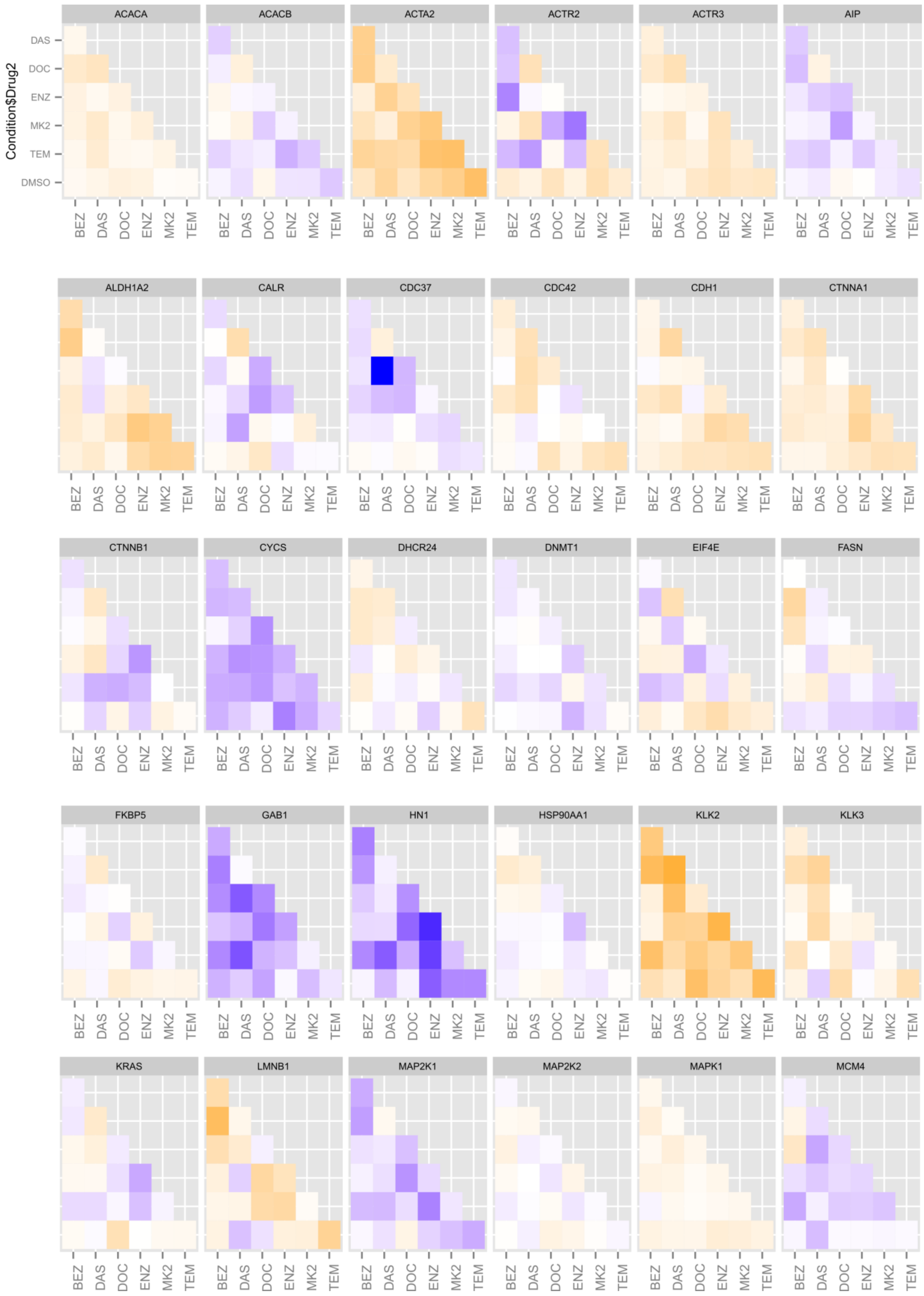
ACACA
ACACB
ACTA2
ACTR2
ACTR3
AIP
ALDH1A2
CALR
CDC37
CDC42
CDH1
CTNNA1
CTNNB1
CYCS
DHCR24
DNMT1
EIF4E
FASN
FKBP5
GAB1
HN1
HSP90AA1
KLK2
KLK3
KRAS
LMNB1
MAP2K1
MAP2K2
MAPK1
MCM4
Condition$Drug2
DAS
DOC
ENZ
MK2
TEM
DMSO
BEZ
DAS
DOC
ENZ
MK2
TEM

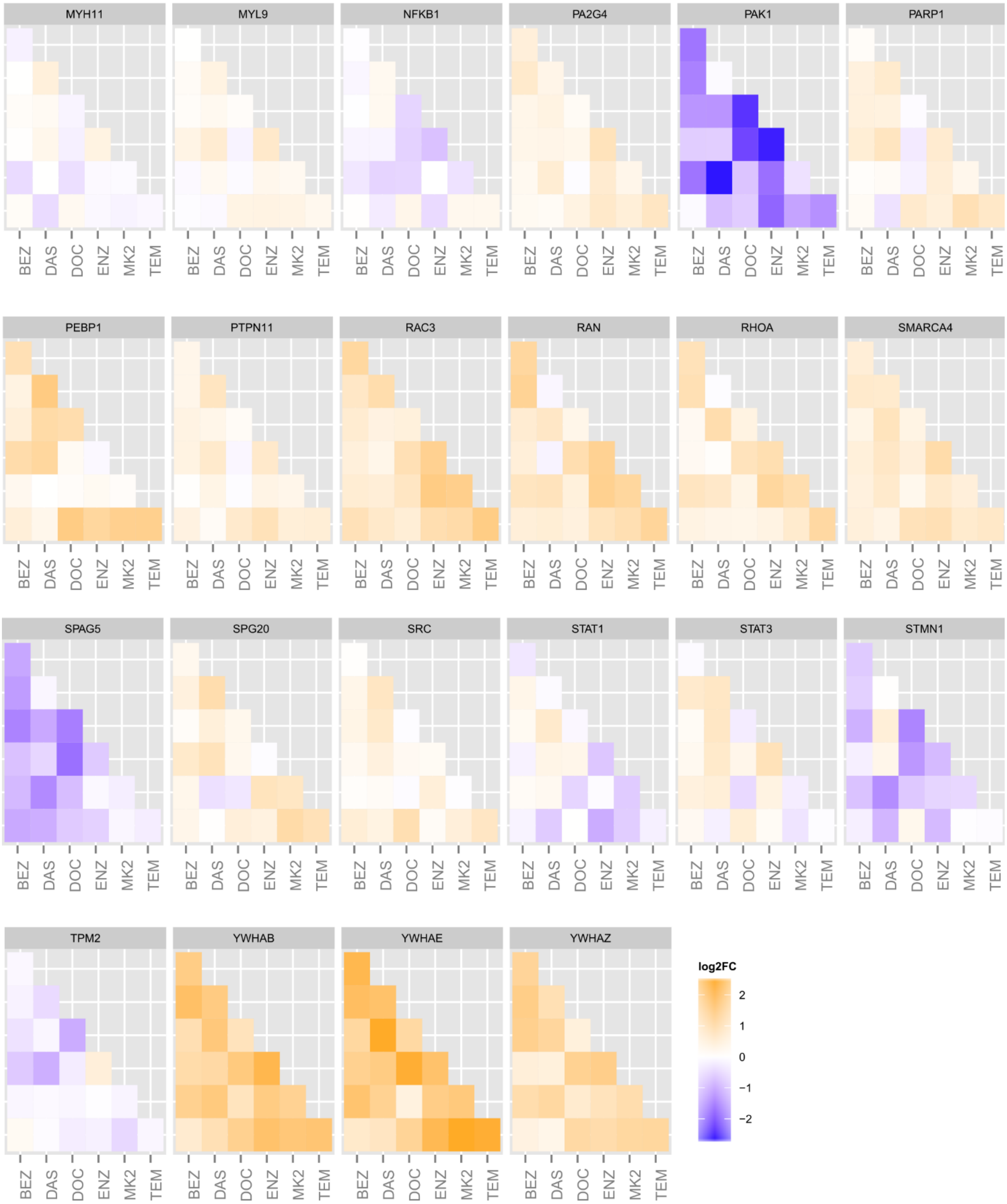

Supplementary Figure 4: SRM data analysis II. Protein centric abundance changes per condition relative to untreated. In these triangle plots the first drug is on the x-axis, while the second drug is along the y-axis. Protein names are official gene names.

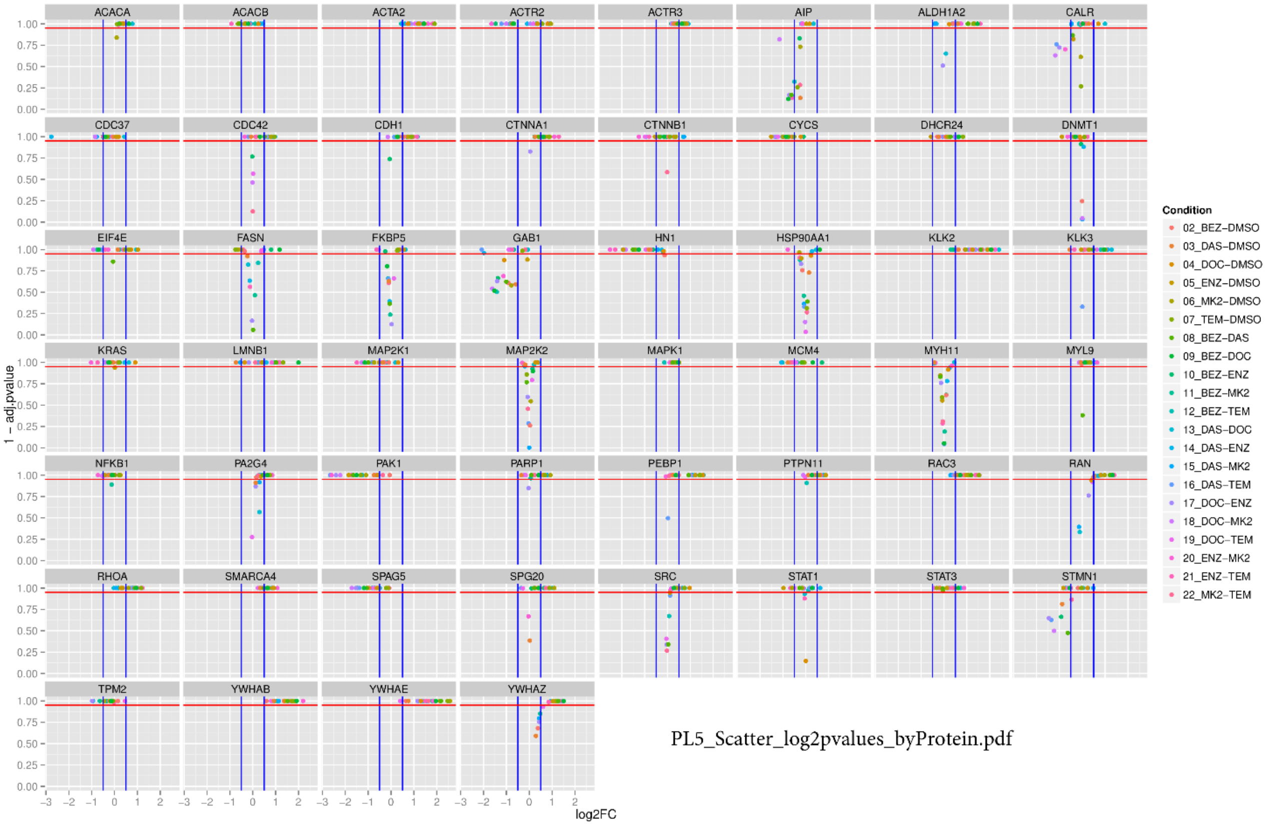

Supplementary Figure 5: SRM data analysis III. A protein centric analysis plotting statistical significance as function of ratio between treatment condition to untreated. Protein names are official gene names.

Cell line	sample name	Drug 1	Concentration drug1	Drug 2	Concentration drug 2	Time point
Lncap	01_DMSO	DMSO	NA	DMSO	NA	24hrs
Lncap	02_BEZ-DMSO	Bez235	500nM	DMSO	NA	24hrs
Lncap	03_DAS-DMSO	Dasatinib	100nM	DMSO	NA	24hrs
Lncap	04_DOC-DMSO	Docetaxel	10nM	DMSO	NA	24hrs
Lncap	05_ENZ-DMSO	Enzalutamide	10µM	DMSO	NA	24hrs
Lncap	06_MK2-DMSO	MK2206	1µM	DMSO	NA	24hrs
Lncap	07_TEM-DMSO	Temsirolimus	100nM	DMSO	NA	24hrs
Lncap	08_BEZ-DAS	Bez235	500nM	Dasatinib	100nM	24hrs
Lncap	09_BEZ-DOC	Bez235	500nM	Docetaxel	10nM	24hrs
Lncap	10_BEZ-ENZ	Bez235	500nM	Enzalutamide	10µM	24hrs
Lncap	11_BEZ-MK2	Bez235	500nM	MK2206	1µM	24hrs
Lncap	12_BEZ-TEM	Bez235	500nM	Temsirolimus	100nM	24hrs
Lncap	13_DAS-DOC	Dasatinib	100nM	Docetaxel	10nM	24hrs
Lncap	14_DAS-ENZ	Dasatinib	100nM	Enzalutamide	10µM	24hrs
Lncap	15_DAS-MK2	Dasatinib	100nM	MK2206	1µM	24hrs
Lncap	16_DAS-TEM	Dasatinib	100nM	Temsirolimus	100nM	24hrs
Lncap	17_DOC-ENZ	Docetaxel	10nM	Enzalutamide	10µM	24hrs
Lncap	18_DOC-MK2	Docetaxel	10nM	MK2206	1µM	24hrs
Lncap	19_DOC-TEM	Docetaxel	10nM	Temsirolimus	100nM	24hrs
Lncap	20_ENZ-MK2	Enzalutamide	10µM	MK2206	1µM	24hrs
Lncap	21_ENZ-TEM	Enzalutamide	10µM	Temsirolimus	100nM	24hrs
Lncap	22_MK2-TEM	MK2206	1µM	Temsirolimus	100nM	24hrs

Supplementary MM 1: Perturbation matrix. List of 21 treatment conditions plus vehicle (DMSO) control with drug concentrations.

**Cell Lysis: Reagents**	**Procedure**
**Lysis buffer**	resuspend cell pellets in 100 µL **Lysis buffer** (up/down pipetting)
71296 | PhosphoSafe™ Extraction Reagent from Millipore	sonicate 1.5 mL tubes with 90% intensity 0.8 cycle time (on/off) 20x
	BCA assay
**Reduction and Alkylation: Reagents**	**Reduction and Alkylation: Procedure**
0.2 M TCEP in 0.1 M HEPES (stored @ -20°C)	Add 4 µL of 0.2 M TCEP
Tris(2-carboxyethyl)phosphine hydrochloride	Incubate for 15 min @ 37 °C on a shaker with 1,400 rpm
MW 286.65 g/mol = 29 mg in 500 µL	Cool down the sample to room temperature
	Add 4 µL of 0.4 M iodoacetamide
0.4 M iodoacetamide in 0.1 M HEPES	Incubate for 30 min @ 25 °C on a shaker with 1,400 rpm in the dark
MW 184.96 g/mol = 37 mg in 500 µL	Spin down 5 min at 12,000 rcf @ 20 °C
**Trypsin digest: Reagents**	**Trypsin digest: Procedure**
Trypsin, sequencing grade modified	Add 3 µL 1 M NaOH, Check pH
V511C, Promega; stored @ -80 °C	add trypsin (2 µL or 1:100 dil.)
aliquots of 20 µg (40 µL, 0.5 µg/µL)	incubate overnight at 37 °C and 250 rpm (floor shaker)
**SDS removal: Reagents**	**SDS removal: Procedure**
Detergent Removal Resin, Columns	remove bottom plug (top only after first spin, resin inside!)
(Thermo Scientific, 877777)	centrifuge for 1 min @ 1'500 rcf
SAMPLE VOLUME 25 - 250 µL	add 0.4 mL 0.1 M HEPES 3x
	place in new collection tube, add 25 -250 µL sample (1:1 ratio)
	**close lid, vortex, incubate for 10 min at room temp**
	centrifuge for 2 min @ 1'500 rcf
	collect SDS free peptides
**C18 cleanup: Reagents**	**C18 cleanup: Procedure**
Sep-Pak Vac 1cc (50 mg) WAT036820 tC18 Cartridges	Adjust pH to 2-3 using 10 % TFA (approx 8 µL)
**Wash C18**	apply supernatante to C18
5 % acetonitrile	1 mL acetonitrile VAC
0.1 % trifluoroacetic acid (TFA)	1 mL **Wash C18** VAC 3x
	Apply sample vac OFF
**Eulte C18**	1 mL **Wash C18** VAC 3x
50 % acetonitrile	0.5 mL **Elute C18** syringe
0.1 % trifluoroacetic acid (TFA)	DRY under vaccum 45 °C
**HILIC**	**HILIC**
Harvard Apparatus GmbH	add 400 µL 100 % methanol
Macro SpinColumns, Hydrophilic Packing Material	centrifuge for 2 min @ 1100 rcf
Qty. of 96; 744305	discard flowthrough
	add 400 µL **dH$_2$O**
**Conditioning Buffer pH 4**	centrifuge for 1 min @ 1100 rcf 3x
0.2 M monosodium acetate,	discard flowthrough
0.3 M sodium acetate	add 600 µL **Conditioning Buffer pH 4**
	centrifuge for 30 sec. @ 1100 rcf
**Loading Buffer pH 6**	**let equilibrate for 1 hour**
25 mM ammonium formate	centrifuge for 1 min @ 1100 rcf
80 % acetonitrile	add 400 µL **Loading Buffer pH 6**
	centrifuge for 1 min @ 1100 rcf 3x
	discard flowthrough
**Elute HILIC**	resuspend dried peptides in 400 µL **Loading Buffer pH 6**
2 % formic acid, HPLC water	sonicate sample, 5 min
	centrifuge for 1 min @ 1100 rcf
	discard flowthrough, add HILIC columns to a new 2 mL tube
	add 400 µL **Elute HILIC**
	centrifuge for 2 min @ 1100 rcf
	dry under vaccum 45 °C

Supplementary MM 2: Peptide generation protocol. Step-by-step protocol, including reagents used, to generate purified peptides free of neutral lipids.
